# Impaired peroxisomal beta-oxidation in microglia triggers oxidative stress and impacts neurons and oligodendrocytes

**DOI:** 10.3389/fnmol.2025.1542938

**Published:** 2025-01-30

**Authors:** Ali Tawbeh, Catherine Gondcaille, Fatima-Ezzahra Saih, Quentin Raas, Damien Loichot, Yannick Hamon, Céline Keime, Alexandre Benani, Francesca Di Cara, Mustapha Cherkaoui-Malki, Pierre Andreoletti, Stéphane Savary

**Affiliations:** ^1^Centre des Sciences du Goût et de l'Alimentation, CNRS, INRAE, Institut Agro, University of Bourgogne, Dijon, France; ^2^Aix Marseille Univ, CNRS, INSERM, CIML, Marseille, France; ^3^Plateforme GenomEast, IGBMC, CNRS UMR, Inserm, University of Strasbourg, Strasbourg, France; ^4^Department of Microbiology and Immunology, IWK Health Centre, Dalhousie University, Halifax, NS, Canada

**Keywords:** neuroinflammation, peroxisome, microglia, oxidative stress, reactive oxygen species, nitric oxide, X-linked adrenoleukodystrophy

## Abstract

Microglia, the immune cells of the central nervous system, activate neuroinflammatory pathways in response to homeostatic disturbances, a process implicated in the pathogenesis of various neurodegenerative diseases. Emerging evidence identifies abnormal microglial activation as a causal factor at the onset of peroxisomal leukodystrophies, including X-linked adrenoleukodystrophy (X-ALD). This study investigates how primary peroxisomal deficiencies influence oxidative properties of microglia and examines the subsequent impact on neurons and oligodendrocytes. Using BV-2 microglial cells lacking ABCD1, ABCD2, or ACOX1, peroxisomal proteins that play key roles in the very-long-chain fatty acid beta-oxidation, we analyzed their response under basal condition and after stimulation by lipopolysaccharide (LPS). Transcriptomic analysis of the mutant microglial cells revealed numerous differentially expressed genes, particularly in redox-related pathways following LPS exposure. These changes are consistent with the increased production of reactive oxygen species (ROS) and nitric oxide (NO). Conditioned media (CM) from the mutant cells were then applied to cultures of neuron and oligodendrocyte cell lines. Exposure to CM from LPS-stimulated mutant microglial cells significantly increased apoptosis in both cell types. Furthermore, treated neurons exhibited a reduction in cell complexity and an increased ability to secrete neuropeptides. These findings demonstrate that peroxisomal impairments in microglia exacerbate inflammatory response and ROS/NO production, affecting the survival of neurons and oligodendrocytes, as well as neuronal morphology and function. This dysfunction might contribute to the early neurodegenerative events in X-ALD by triggering and sustaining neuroinflammatory cascades. Therapeutic strategies that target microglial activation and secretion profiles could hold promise in managing peroxisomal disorders such as X-ALD.

## Introduction

Microglia, the primary immune cells of the central nervous system, play essential roles in maintaining brain homeostasis, modulating neural activity and synaptic plasticity, and contributing to normal brain development and function (Wright-Jin and Gutmann, 2019). Disruptions in microglial homeostatic state have been associated with neuroinflammation in various neurological disorders (Sirkis et al., 2021; Paolicelli et al., 2022). Microglia mediate host defense against infectious pathogens and injurious self-proteins by initiating a neuroinflammatory response, which includes the production of (NO) through the inducible nitric oxide synthase (NOS2) and cytokines such as tumor necrosis factor-alpha (TNF) and interleukin-1β (IL1B). However, persistent neuroinflammation induces neurotoxicity, which consequently leads to neurodegeneration (Hickman et al., 2018).

There is ample evidence documenting the activation of microglia in neurodegenerative disorders, leading to the overproduction of reactive oxygen species (ROS) and NO (Banati et al., 1993; Gehrmann et al., 1995; Hopkins and Rothwell, 1995). The most common neurodegenerative diseases, such as Alzheimer’s disease, Parkinson’s disease, Huntington’s disease, and amyotrophic lateral sclerosis, are all characterized by microglia-mediated neuroinflammation and increased oxidative stress (Wu and Zou, 2022). Under physiological circumstances, these immune mediators can function as scavengers, aiding in the elimination of necrotic neurons and managing their regular turnover. However, in neurodegenerative diseases, persistently activated microglia may worsen the condition by excessively releasing these cytotoxic factors.

Peroxisomes, organelles found in eukaryotic cells, play key roles in lipid metabolism and cellular redox balance (Wanders et al., 2023). Their crucial roles in regulating immune and inflammatory responses have been highlighted over the past few years (Di Cara et al., 2019; Di Cara, 2020; Di Cara et al., 2023). However, research focusing on investigating the relationship between peroxisomes and microglia is missing. To fill this gap, we established knockout (KO) BV-2 cell lines for the peroxisomal ABC transporters of very long-chain fatty acids (VLCFA), ABCD1 and ABCD2 (Raas et al., 2019a; Tawbeh et al., 2021), and for the rate-limiting enzyme of peroxisomal *β*-oxidation, ACOX1 (Raas et al., 2019b). These KO BV-2 cells constitute cell models for X-linked adrenoleukodystrophy (X-ALD, MIM 300100), the most common peroxisomal disorder, which is linked to mutations in the *ABCD1* gene (Mosser et al., 1993; Trompier and Savary, 2013; Kemp et al., 2016), and for a rarer disease, ACOX1-deficiency (MIM264470) (Poll-The et al., 1988; Ferdinandusse et al., 2007). Using these cell models, we previously demonstrated that dysfunctional peroxisomal *β*-oxidation of fatty acids in microglia induces a transcriptomic signature resembling the disease-associated microglia (DAM) signature (Raas et al., 2023). DAM state is characterized by the repression of several homeostatic genes, such as *Cx3cr1* and *P2ry12*, and the upregulation of various genes including *Trem2, Apoe*, or *Ctsb.* Interestingly, elevated levels of cathepsin B (CTSB), one of the proteins we found oversecreted in the medium of the KO BV-2 cells, were shown to induce neuroinflammation and trigger neurodegeneration (Nakanishi, 2020; Pislar et al., 2021). In addition, using mutant BV-2 cells, we demonstrated that defects in peroxisomal *β*-oxidation lead to oversecretion of pro-inflammatory cytokines, particularly TNF, upon stimulation with lipopolysaccharide (LPS) (Tawbeh et al., 2023). Increased TNF levels have also been associated with neuronal cell death in neurodegenerative diseases such as Alzheimer’s disease (Jayaraman et al., 2021). An excessive response to LPS along with the repression of homeostatic microglial genes, has also been reported *in vivo* using a KO mouse model of the *Hsd17b4* gene, which encodes MFP-2, the second enzyme in the peroxisomal β-oxidation pathway (Verheijden et al., 2014; Beckers et al., 2019).

Peroxisomal dysfunction in microglia has been proposed to trigger neurodegenerative cascades in X-ALD (Gong et al., 2017; Bergner et al., 2019) and oxidative stress is considered as a key driver of neurodegeneration (Yu et al., 2022). ROS levels are maintained within a state of dynamic equilibrium via a confluence of ROS-generating processes and cellular defense mechanisms aimed at counteracting their deleterious effects. Oxidative stress occurs when ROS production exceeds the capacity of the body’s antioxidant defense systems, resulting in cellular damage (Salim, 2017). Microglia, acting as phagocytes, have the ability to trigger an “oxidative/respiratory burst” as a response to damaging molecules and substances associated with pathogens, such as aggregated proteins and cellular debris (Claude et al., 2013; Herzog et al., 2019; Simpson and Oliver, 2020). In addition to ROS, NO is a signaling molecule involved in a large number of physiological functions and can be harmful when overproduced. High concentrations of NO, along with ROS, are considered key triggers of neurodegenerative diseases (Yuste et al., 2015).

Thus, it was of particular interest to explore the redox phenotype and the expression levels of oxidative stress-related genes in wild-type (WT) and peroxisomal mutant BV-2 cells, in basal condition and during activation via LPS stimulation. Aiming to investigate the functional consequences of the secretions of microglia with peroxisomal dysfunction on neurons and oligodendrocytes, we also implemented coculture and conditioned media (CM) experiments using the BV-2 cells. The results showing that cell survival, morphology, and function are affected provide an *in vitro* insight into the pathogenesis underlying the neurodegeneration in X-ALD.

## Materials and methods

### Resource availability

The data discussed in this publication have been deposited in NCBI’s Gene Expression Omnibus (Edgar et al., 2002) and are accessible through GEO Series accession number GSE200022^1^ for the genome comparison and GSE237635 for the LPS effect. The data supporting the findings and the Excel files containing the gene lists used to create the figures are available from the corresponding author (SS) upon request.

### RNA-sequencing and differential gene expression analysis

All the procedures describing the RNA sample preparation, the RNA-seq library preparation, sequencing, and the differential gene expression analysis were previously detailed (Raas et al., 2023; Tawbeh et al., 2023). Briefly, total RNA was extracted from three independent batches of BV-2 cells for each genotype (WT, *Abcd1^−/−^Abcd2^−/−^*, and *Acox1^−/−^*) using the RNeasy kit (Qiagen). RNA-sequencing libraries were prepared using the Illumina TruSeq Stranded mRNA LT Sample Preparation Kit, and sequencing was performed on the Illumina HiSeq4000 sequencer as single-end 1×50 base reads. Differential gene expression analyses were performed using R 3.3.2 and DESeq2 version 1.16.1 Bioconductor library (Love et al., 2014). To analyze the effect of the LPS treatment (24 h with 1 μg/mL LPS from *Escherichia coli* O55:B5 (Sigma-Aldrich)), we defined a model with one factor (the corresponding condition). To test if the treatment effect differs between two genotypes, we defined a model with two factors (genotype and treatment) and their interaction. Wald test *p*-values were adjusted for multiple testing using the Benjamini and Hochberg method (Benjamini and Hochberg, 1995). DEGs were selected using the following thresholds: adjusted *p*-value lower than 0.05 and absolute log2 fold change (FC) higher than 1. Venn diagrams were obtained using Venny 2.1^2^.

### Cell culture and treatments

The normal and mutant BV-2 cell lines (WT, *Abcd1^−/−^Abcd2^−/−^*, and *Acox1^−/−^*) were used and cultured as described previously in (Raas et al., 2019a; Raas et al., 2019b). The murine mHypoA-POMC/GFP-1 cell line, which is an immortalized adult-derived neuronal POMC-expressing/*α*-MSH-secreting cell model (Nazarians-Armavil et al., 2014), was purchased from Cedarlane Labs (CLU-500). The murine 158 N cell line, an immortalized oligodendrocytes cell line, was created and provided by Said Ghandour CNRS-ER 2072, Institut de Chimie Biologique, Faculté de Médecine, Strasbourg, France (Feutz et al., 2001). All these cell lines were cultured in the same medium, DMEM supplemented with 10% heat-inactivated FBS, 100 U/mL penicillin, 100 μg/mL streptomycin, and incubated at 37°C, 5% CO_2_. For each experiment, cells were seeded to reach 70–90% confluency in the control condition at the end of the treatment period (unless otherwise noted).

LPS treatment of BV-2 cells was performed at a concentration of 1 μg/mL using LPS from *Escherichia coli* serotype O55:B5 (Sigma-Aldrich L2880). The treatment duration is specified below.

Recombinant mouse TNF (R&D Systems 410-MT-01/CF), NO donor S-Nitro-N-acetylpenicillamine (SNAP—Sigma-Aldrich N3398), and CTSB (Acro Biosystems CTB-M52H9) treatments of mHypoA and 158 N cells were performed in a 24-well plate for 48 h.

Regarding TNF, we used concentrations reflecting the concentration measured in the supernatant of LPS-treated BV-2 WT cells (0.447 ng/mL) and mutant BV-2 cells (1.26 ng/mL) (Tawbeh et al., 2023). The chosen SNAP concentrations replicate the NO concentrations observed in the secretome of LPS-treated mutant cells based on prior calibration experiments. We used 50 μM SNAP to achieve an NO concentration of 15 μM (matching the levels from *Abcd1^−/−^Abcd2^−/−^* cells) and 6 μM SNAP to achieve an NO concentration of 2.5 μM (matching the levels from *Acox1^−/−^* cells). Cathepsin B treatments were performed at 155 ng/mL, 460 ng/mL, or 2,518 ng/mL, corresponding to the levels measured in the supernatants of WT, *Abcd1^−/−^Abcd2^−/−^*, and *Acox1^−/−^* BV-2 cells, respectively (Raas et al., 2023).

### Conditioned media preparation

CM were prepared from normal and mutant BV-2 cells treated with LPS or exposed to myelin sheath debris. BV-2 cells were cultured in 6-well plates for 24 h and then treated or not treated with LPS for an additional 24 h. At the end of the treatment, the CM were collected and centrifuged at 300 g for 7 min to eliminate remaining cells or debris and stocked at −80°C until used. To obtain myelin-exposed BV-2 CM, BV-2 cells were seeded in 96-well plates for 24 h, then incubated for 24 h with 10 μg/mL myelin sheath debris prepared as described previously (Tawbeh et al., 2023). After the treatment, the supernatants were recovered, centrifuged at 1,000 g for 10 min to remove myelin debris from the medium, and stocked at −80° C until used.

### Viability assay and cell death characterization

To determine the effect of LPS-treated BV-2 CM on cell viability, 20,000 mHypoA or 158 N cells were seeded in 1 mL of CM per well in a 24-well plate. The CM from BV-2 exposed to myelin sheath debris were tested seeding 5,000 mHypoA cells in 200 μL of CM per well in 96-well plates. Cell viability was determined by MTT assay 48 h after the seeding. MTT test is based on the reduction of the MTT tetrazolium salt (3-(4,5-dimethylthiazol-2-yl)-2,5-diphenyl tetrazolium bromide) to formazan by mitochondrial succinate dehydrogenase in living cells. The quantity of formazan produced is proportional to the metabolic activity of cells. This insoluble formazan crystal is solubilized in dimethyl sulfoxide (DMSO) to enable spectrophotometric determination. In practice, at the end of the culture, the supernatants were replaced with a complete medium containing 1 mg/mL MTT (Sigma-Aldrich), and cells were incubated at 37°C and 5% CO_2_ for 3 h. The medium was then removed, and DMSO was added (500 μL for 24-well plates or 100 μL for 96-well plate). The absorbance of the assay solution was measured at 570 nm using a microplate reader (TECAN M200 Infinite Pro).

To characterize the cell death induced by LPS-treated BV-2 CM, that is, to determine the percentage of apoptotic or necrotic cells in each condition, Annexin V-FITC and propidium iodide (PI) staining was performed using the “Annexin V-FITC/PI Apoptosis Detection Kit” (Cohesion Biosciences, reference CAK2001). For this purpose, after culturing in CM, cells were trypsinized, washed with cold DPBS, and resuspended in binding buffer at a density of 3×10^6^ cells/mL. A 100 μL suspension was then stained with 5 μL Annexin V-FITC, incubated for 5 min at room temperature in the dark, and then stained with 10 μL PI. After 5 min of incubation, 200 μL DPBS was added, and the resulting suspension was homogenized gently and subsequently analyzed using an LSRII Becton Dickinson Flow cytometer. For each sample, 10,000 events were captured, and the data analysis was performed using FlowJo V10 software. Cells were categorized based on their fluorescence labeling as follows: alive (FITC^−^/IP^−^), necrotic (FITC^−^/IP^+^), early apoptotic (FITC^+^/IP^−^), or late apoptotic (FITC^+^/IP^+^).

### Sholl analysis of neuronal morphology

Sholl analysis is a quantitative method used to characterize the morphologic parameters of neurons. For this purpose, neurons are stained and imaged. Neurons were seeded at 20,000 cells/mL on circular 10-mm diameter glass coverslips in 24-well plates in LPS-treated BV-2 CM and cultured for 48 h. Fluorescent immunocytochemistry was performed on neurons following fixation with PFA 4% for 10 min, permeabilization with Triton X-100 0.1% for 10 min, and blocking with 5% mouse serum for 20 min, including PBS wash between each step. Neurons were stained with a global neurite marker, mouse anti-MAP2ab antibody (Arigo Biolaboratories ARG52328), diluted 1:200 in PBS containing 0.05% saponin and 2% mouse serum, incubated for 40 min then washed with PBS containing 0.05% saponin. Afterward, the samples were incubated with the secondary antibody, AlexaFluor 546 goat anti-chicken antibody (Invitrogen), which was diluted 1:1,000 in PBS, 0.05% saponin, and 2% mouse serum. After the last wash, coverslips were mounted in a fluorescent preservative medium.

The stained cells were observed at ×20 magnification using a Zeiss Axio Imager M2 epifluorescence microscope, equipped with an AxioCamMRm CCD camera and Axiovision software v4.8.2 (Carl Zeiss). Photos were taken x20 mosaic option with the groups being blinded. Four different images were taken in 4–5 different zones. Positions are chosen randomly but with preference to those showing individualized neurons. From each image, 2–4 neurons were analyzed to have a total of 10–12 neurons studied at each condition. Two biological replicates were conducted obtaining a total of 20–22 isolated neurons. Neurite outgrowth was assessed using Fiji software v2.3.0 (Schindelin et al., 2012), and the following parameters were examined: the number of neurite branches, neuronal length, and Sholl profile (Sholl, 1953). Neuronal length was defined as the total length measured between the farthest extremities of a neuron. To generate the Sholl profile, the number of neurite branches was plotted against the distance from the cell soma. Graphs were plotted using GraphPad Prism version 9.4.1.

### Intracellular ROS production in BV-2 cells

Oxidative stress was assessed using a chloromethyl derivative of 2′,7′-dichlorodihydrofluorescein (CM-H_2_DCFDA, ThermoFisher) and dihydroethidium (DHE). H_2_DCFDA and DHE are membrane-permeable molecules that are oxidized by intracellular ROS that produce fluorescent DCF and ethidium, respectively. CM-H_2_DCFDA offers better intracellular retention than H_2_DCFDA, and its chloromethyl group is thiols reactive. BV-2 cells seeded in a 96-well plate were treated with LPS for 3–24 h. Following the treatment, the culture medium was removed and cells were washed one time with prewarmed DPBS and then incubated with either CM-H_2_DCFDA (5 μM) for 70 min or DHE (10 μM) for 30 min at 37°C in 5% CO_2_ atmosphere. Using a plate reader (TECAN infinite M200Pro), the fluorescence was then directly measured at 485 nm or 518 nm for excitation and 535 nm or 605 nm for emission to detect DCF and ethidium, respectively. For the ROS basal production comparison, as mutant and WT BV-2 cells have different growing kinetics, ROS production was standardized to cell quantity, which was evaluated using Hoechst 33342. For this purpose, in identical but distinct wells, cells were treated and dyed in the same manner with Hoechst 33342 (3.5 μM) for 10 min. Fluorescence was measured at 350 nm for excitation and 461 nm for emission. For each experiment, values were calculated as the ROS dye fluorescence mean of a triplicate normalized to the corresponding Hoechst fluorescence mean.

### NO production in BV-2 cells

BV-2 cells seeded in a 6-well plate were treated with LPS for 7 h or 24 h. The supernatant was collected. Cells were washed with DPBS and homogenized in solubilization buffer (100 mM Tris–HCl, pH 8, 100 mM NaCl, 10 mM EDTA, 1% Triton X-100) containing 1% PMSF and protease inhibitor mixture (Roche Applied Science). Nuclei were removed by centrifugation for 10 min at 4°C at 1,000 g, and the cellular protein extract was collected. Protein concentration was quantified using the BiCinchoninic Acid (BCA) kit (Sigma-Aldrich) according to the manufacturer’s instructions. The production of BV-2 nitrite, the final product of NO oxidation, was assessed in the supernatant by the Griess reaction in a 96-well plate in triplicate: 50 μL of supernatant were mixed with an equal volume of Griess reagent (0.1% N-1-naphtylethylenediamine dihydrochloride and 1% sulfanilamide in 5% phosphoric acid—Sigma-Aldrich) and incubated for 15 min at room temperature. The absorbance was measured at 540 nm using a plate reader (TECAN Sunrise). The concentration of nitrite in the sample was calculated from a standard curve obtained from solutions of sodium nitrite in a culture medium ranging from 0 to 100 μM. Nitrite quantity was reported to protein quantity per well.

### Secretion of *α*-MSH by mHypoA neurons

mHypoA cells were seeded in 6-well plates at a density of 70,000 cells per well in 3 mL LPS-treated BV-2 CM. After 48 h in culture, the supernatant was removed, cells were washed once with DPBS, and 1.5 mL serum-free DMEM was added to the cells that were cultured for an additional 4 h. To induce the secretion of alpha-melanocyte-stimulating hormone (α-MSH), the medium was replaced with serum-free DMEM containing 60 mM KCl (1.5 mL per well), and the cells were cultured for an additional 20 min. The media were then collected as 500 μL aliquots and transferred to the SpeedVac concentrator until the total evaporation of the supernatants (approximately 8 h). Afterward, the samples were kept at −80°C until the day of analysis. Samples were collected in triplicate for each experimental repeat. This protocol was published by Nazarians-Armavil et al. (2014), with further details obtained after contacting their laboratory. α-MSH secretion was determined using the α-MSH (Human, Rat, Mouse) enzyme immune assay (EIA) kit (Phoenix Pharmaceuticals, Inc. EK-043-01). For this assay, the dried samples were rehydrated in 50 μL assay buffer, and the totality of the sample was loaded onto the assay plate according to the manufacturers’ instructions. Absorbance measurements were performed on the TECAN M200 Infinite Pro microplate reader. *α*-MSH secretion values were normalized to 1/3 of the total number of cells per well, as 500 μL were aliquoted out of the 1,500 μL, which is the volume of each well. The results were expressed as α-MSH (pg) per 100,000 cells.

### Catalase activity

Catalase activity was assessed by photometric measurement of cell extract H_2_O_2_ consumption. BV-2 cells seeded in a 6-well plate were treated with LPS for 7 h or 24 h. The cellular extract and its protein quantification were performed as described above. Catalase activity was measured in a 96-well plate, with cell extract diluted 20-fold in 50 mM Tris–HCl, pH 7.4, and 20 mM H_2_O_2_. The decreasing rate of H_2_O_2_ absorbance at 240 nm was monitored for 2 min using a plate reader (TECAN infinite M200Pro). The catalase-specific activity was calculated as the H_2_O_2_ concentration variation per min reported to the protein quantity and was expressed as μmol/min/mg of protein.

### Western blotting analysis

Western blotting analysis was performed as described previously (Tawbeh et al., 2023). The following antibodies were used with the indicated dilution: anti-arginase 1 (ARG1, 1:1,000, Genetex 109,242), anti-catalase (CAT, 1:400, R&D Systems AF3398), anti-glutathione reductase (GSR, 1:1,000, Abcam ab137513), anti-inducible nitric oxide synthase (NOS2, 1:1,000, Abcam ab136918), anti-NADPH oxidase 1 (NOX1, 1:1,000, Abcam ab131088), and anti-peroxiredoxin 5 (PRDX5, 1:200, R&D Systems AF5724). Protein amount loading control and normalization were achieved by probing the membranes with α-tubulin (TUBA1A, 1:4,000, Sigma-Merck T5168) or *β*-actin (ACTB, 1:10,000, Sigma-Merck A5441) antibodies.

### Statistical analyses

The different statistical tests applied are indicated in the figure legends. Statistical analyses were conducted using GraphPad Prism 9. All results were presented as mean ± standard error of the mean (SEM) or standard deviation (SD), as indicated in the figures. Statistical analyses were performed using *t*-tests for comparisons between two groups, one-way ANOVA for comparisons among multiple groups with one independent variable, and two-way ANOVA for comparisons involving multiple groups with two independent variables.

## Results

### Oxidative stress markers in BV-2 mutant microglial cells

#### Transcriptomic data suggest an overresponse to LPS treatment

**Figure 1:**
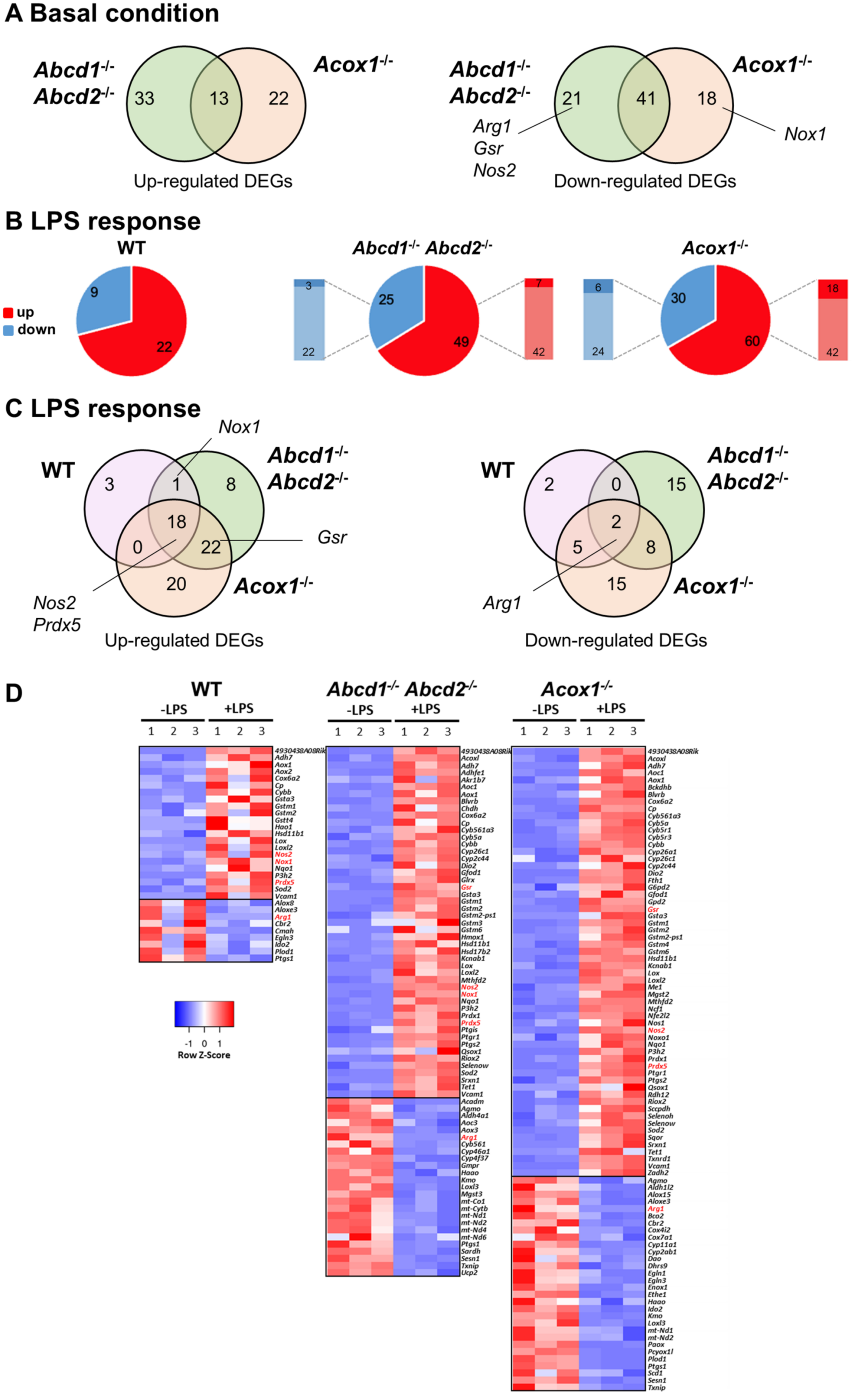
Transcriptomic analysis of redox-associated genes and their LPS response in WT and mutant (*Abcd1*^−/−^*Abcd*2^−/−^ and *Acox1*^−/−^) BV-2 cells (*n* = 3 for each genotype). **(A)** Venn diagrams illustrating the up- and downregulated differentially expressed genes (DEGs) involved in the oxidation–reduction process (log2FC > 1, AdjP value <0.05) in non-stimulated *Abcd1*^−/−^*Abcd2*^−/−^ and *Acox1*^−/−^ BV-2 cells than in WT cells (DataSet GSE200022). Genes whose expression level was verified at the protein level are noted. **(B)** Pie chart displaying the number of up- or downregulated DEGs in each genotype after a 24-h LPS treatment (DataSet GSE237635). The significant genes were selected using a cutoff adjusted *p*-value (DESeq2 Wald test with Benjamini and Hochberg *p*-value adjustment) lower than 0.05 and an absolute log2 FC higher than 1. Additional comparison to test if the effect of the LPS treatment is different between the mutant and the WT genotypes is indicated in the left and right bars, with the proportion of DEGs significantly (AdjP value <0.05) more repressed (dark blue) (log2FC < 1) or induced (dark red) (log2FC > 1), respectively. **(C)** Comparative analysis of the sets of DEGs using a Venn diagram illustrating the larger increase of upregulated (up) and downregulated (down) DEGs in LPS-treated mutant cells than in WT cells. **(D)** Heat map representing RNA-seq gene expression (with Z-scores) of these DEGs at 24 h after LPS stimulation in WT, *Abcd1*^−/−^*Abcd*2^−/−^, and *Acox1*^−/−^ BV-2 cells (three independent batches of BV-2 cells for each genotype). Genes whose expression level was verified at the protein level are in red.

Thousands of differentially expressed genes (DEGs) have previously been identified in the mutant BV-2 cells under both basal conditions and after LPS stimulation (Raas et al., 2023; Tawbeh et al., 2023). Gene ontology enrichment analysis had highlighted various pathways linked to immune function, lipid metabolism, and cellular redox balance. Here, we focused on genes related to the “oxidation–reduction process.” The main genes involved in redox control in microglia, which is intimately linked to the function of microglia and has consequences on their environment, have been reviewed extensively (Rojo et al., 2014; Vilhardt et al., 2017; Fan et al., 2023). In basal conditions, we evidenced 49 and 35 upregulated DEGs (13 DEGs belonging to the intersection) in the *Abcd1^−/−^Abcd2^−/−^* and *Acox1^−/−^* genotypes, respectively. Regarding the downregulated DEGs, 62 and 59 (with 41 genes found in the intersection) were found in the *Abcd1^−/−^Abcd2^−/−^* and *Acox1^−/−^* genotypes, respectively (Figure 1A). *Nos2*, the gene encoding inducible nitric oxide synthase (NOS2), which is known as the main provider of NO in microglia; *Arg*1, the gene encoding arginase 1 (ARG1), which inhibits NO production and counteracts nitrosative stress; and *Gsr*, the gene encoding discard glutathione reductase (GSR), a key enzyme of glutathione homeostasis, were found among the repressed DEGs in the *Abcd1^−/−^Abcd2^−/−^* cells. In the *Acox1^−/−^* cells, a significant repression of *Gsr* but just below the threshold (log2FC = 0.90) and non-significant inductions of *Arg1* and *Nos2* were observed. The main pro-oxidant genes encoding NADPH oxidoreductases (NOX1, 3, 4, and DUOX1, 2) exhibited very low expression levels, with the exception of *Cybb* (NOX2). Of these genes, only *Duox2* was found in the upregulated DEGs in *Abcd1^−/−^Abcd2^−/−^* cells, and *Nox1* was found in the downregulated DEGs in the *Acox1^−/−^* cells. Remarkably, *Qsox1*, which protects from oxidative stress when overexpressed (Morel et al., 2007) and induces oxidative and ER stress when invalidated (Caillard et al., 2018), was among the genes the most repressed genes in the basal condition in both KO genotypes. Altogether, these data indicate a moderated deregulation of antioxidant defense and of NO homeostasis in basal conditions.

Upon LPS treatment, we previously documented an oversecretion of inflammatory mediators (Tawbeh et al., 2023). Such a response is usually accompanied by oxidative stress leading to ROS and NO production. As shown in Figure 1, LPS treatment triggered transcriptomic changes in agreement with previous studies in BV-2 cells (Das et al., 2015). This includes the upregulation of *Cybb*, *Gsr*, *Nos1*, *Nos2*, *Nox1*, *Sod2* (gene encoding the ROS scavenger superoxide dismutase 2), *Prdx1* and *Prdx5* (genes encoding peroxiredoxins involved in the reduction of hydrogen peroxide and peroxynitrite) and the downregulation of *Arg1* with some discrepancies between the genotypes, mostly associated with threshold issues. For instance, *Gsr* was also upregulated in the WT cells but with a 0.47 log2FC and *Nox1* displayed a 1.95 log2FC in the *Acox1^−/−^* genotype but with an adjusted *p*-value above 0.05. Interestingly, as compared to the WT cells, the response of KO cells was enhanced both in terms of the total number of DEGs (3-fold more) and the fold-change value for a significant number of DEGs (between 12 and 36%) (Figures 1B–D). As an example, *Nos2* was significantly more upregulated in the *Abcd1^−/−^Abcd2^−/−^* cells than in the WT cells, and *Cybb*, *Gsr, Prdx1, Prdx5,* and *Sod2* were significantly more upregulated in the *Acox1^−/−^* cells.

#### Increased ROS production in mutant BV-2 cells is amplified upon LPS activation

**Figure 2:**
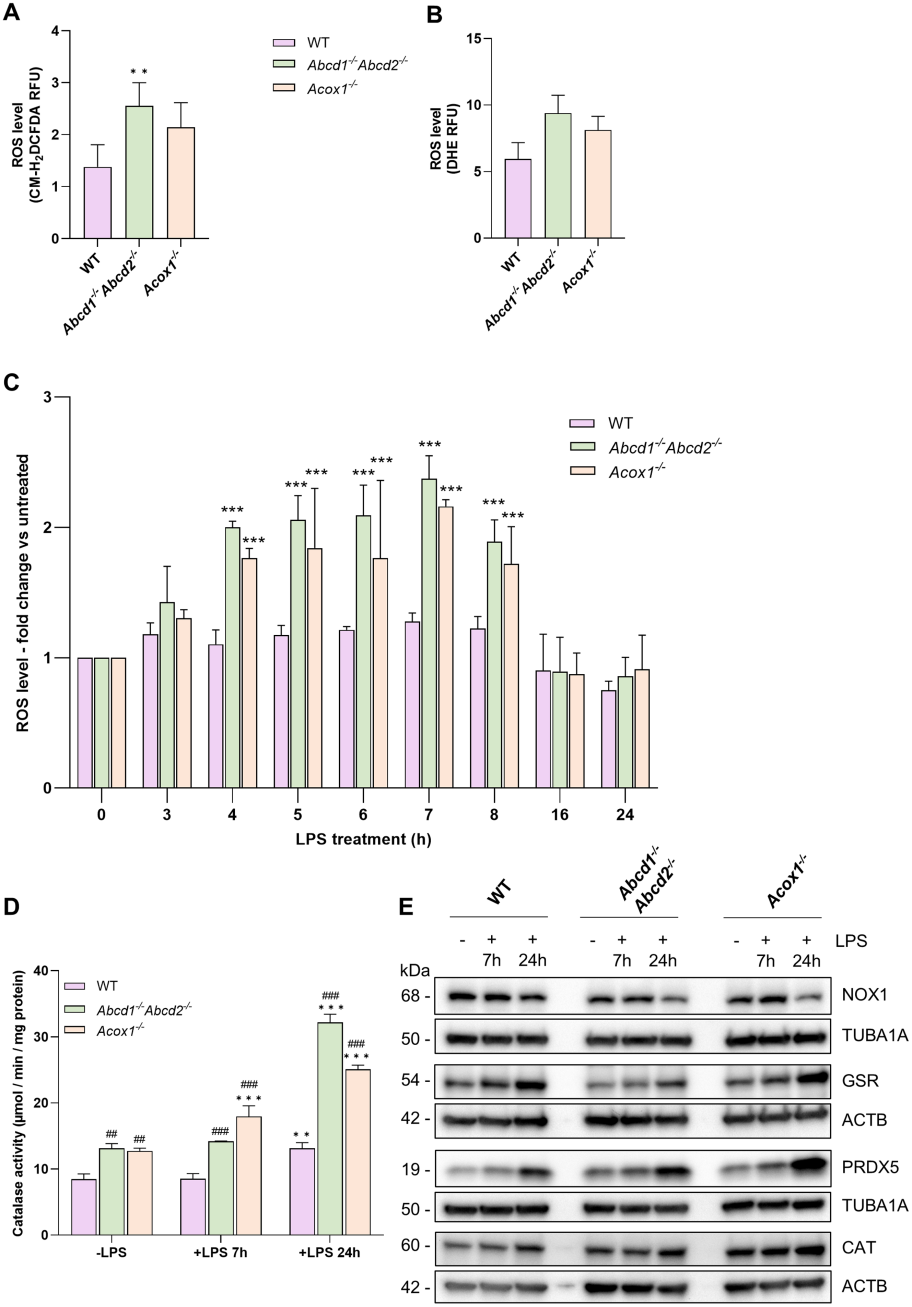
Redox status of WT and mutant BV-2 cells and their LPS response. Intracellular reactive oxygen species (ROS) levels were assessed in both WT and mutant BV-2 cells after 24 h in culture using CM-H2DCFDA **(A)** or dihydroethidium (DHE) **(B)**. ROS fluorescence values were normalized to Hoechst fluorescence to account for cell number. Data represent the means of four independent experiments +SEM. Statistical significance was determined using an unpaired Student’s *t*-test (*p < 0.05, **p < 0.01) for comparisons between mutant and WT BV-2 cells. **(C)** ROS production in response to LPS treatment (1 μg/mL) was monitored over a 24-h period using the CM-H2DCFDA protocol. In each experiment, the triplicate mean fluorescence values of LPS-treated BV-2 cells were compared to those of untreated BV-2 cells. Data are presented as means from three independent experiments +SD, and statistical significance was calculated using two-way ANOVA followed by Dunnett’s multiple comparisons test (*p < 0.05, **p < 0.01, ***p < 0.001) for comparisons between LPS-treated and untreated cells. **(D)** Catalase activity in cell lysates, both in the absence and following 7 or 24 h of LPS treatment, was assessed by measuring the rate of hydrogen peroxide (H_2_O_2_) consumption per minute, normalized to the total protein content. Data represent the means from four independent experiments +SEM. Statistical significance was determined by two-way ANOVA followed by Dunnett’s multiple comparisons test to compare LPS-treated and untreated cells for each genotype (***p < 0.001) and followed by Tukey’s multiple comparisons test to compare KO and WT cells for each treatment condition (##*p* < 0.01, ###*p* < 0.001). **(E)** Representative image of Western blotting analysis (three independent experiments) of redox-related proteins, including NOX1, GSR, PRDX5, and catalase (CAT) in WT, *Abcd1*^−/−^ *Abcd2*^−/−^, and *Acox1*^−/−^ BV-2 cells untreated (−) or treated with LPS for 7 or 24 h (+). Either TUBA1 or ACTB served as loading controls, with the molecular weights of each protein indicated. Source data are available online for this figure.

To complement the results obtained in the transcriptomic analysis indicating oxidative stress-related DEGs, we studied the ROS production in the WT and mutant BV-2 cells in basal conditions and after 24 h of LPS treatment. Using two ROS indicators, CM-H_2_DCFDA (large ROS specificity) (Figure 2A) and DHE (specific for superoxide anion) (Figure 2B), we showed increases in ROS production in both *Abcd1^−/−^Abcd2^−/−^* and *Acox1^−/−^* cells than in WT cells at the basal level, with *Abcd1^−/−^Abcd2^−/−^* cells showing the highest oxidative status (significant induction was only observed with CM-H_2_DCFDA). Using CM-H_2_DCFDA, we investigated the time course of the oxidative response to LPS during 24 h. Unlike the WT cells, which exhibit a moderate response, KO cells, especially the *Abcd1^−/−^Abcd2^−/−^* cells, showed a higher production of ROS with a bell-shaped curve response that peaked at 7 h and returned to baseline levels by 16 h (Figure 2C). This would imply that the redox balance restoration begins shortly after LPS exposure. To further investigate this hypothesis, we measured the activity of catalase, a major player in the peroxisomal antioxidant response. We evidenced a higher activity in the KO cells increasing with time upon LPS treatment (Figure 2D).

In addition, Western blot analyses were conducted to validate at the protein level the data from the RNA-seq analysis (Figure 2E and Supplementary Figure S1 for densitometry analysis). Under basal conditions, KO cells showed a non-significant reduction of GSR and NOX1 expression and an increase in CAT and PDRX5 expression. After LPS treatment (at 7 or 24 h), a time-dependent upregulation of CAT, GSR, and PRDX5 was recorded. The increased variations observed in KO cells were predominantly greater than those in control cells supporting an exaggerated response to LPS. These results are consistent with the transcriptomic data with the exception of NOX1, whose expression at the protein level was unexpectedly downregulated after 24 h incubation with LPS in all three genotypes.

#### Increased NO production in LPS-treated mutant BV-2 cells

**Figure 3:**
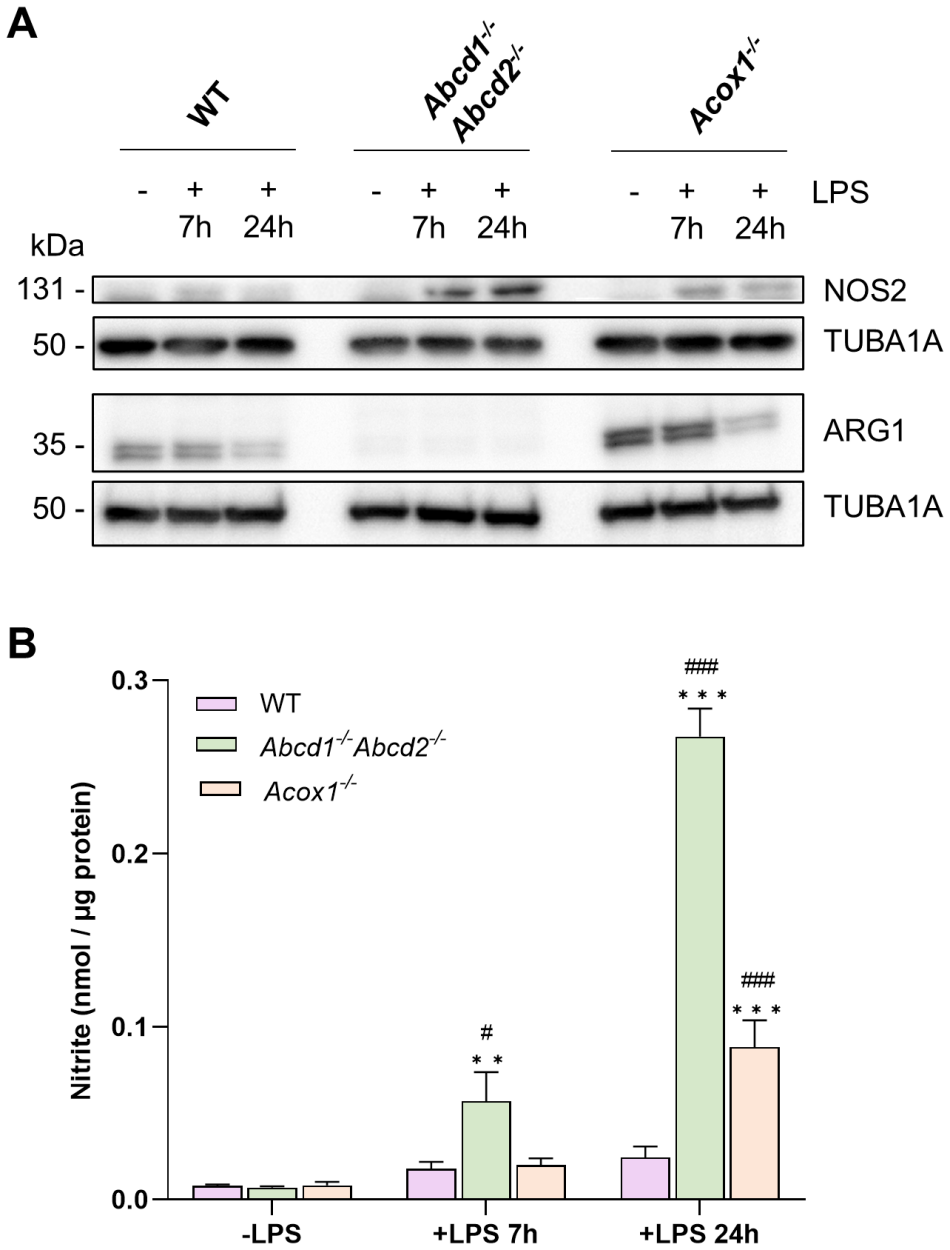
Expression of ARG1 and NOS2 and NO production. **(A)** Representative image of Western blotting analysis (three independent experiments) of the expression levels of ARG1 and NOS2, two main regulators of NO production, in WT, *Abcd1*^−/−^ *Abcd2*^−/−^, and *Acox1*^−/−^ BV-2 cells untreated (−) or treated with LPS for 7 or 24 h (+). TUBA1 served as loading control. Expected molecular weights are indicated. Source data are available online for this figure. **(B)** Nitrite level reported to protein quantity in the medium of WT, *Abcd1*^−/−^ *Abcd2*^−/−^, and *Acox1*^−/−^ BV-2 cells untreated (−) or treated with LPS for 7 or 24 h (+). Nitrites were quantified by Griess reaction. Data represent the mean values of four independent experiments +SEM. Statistical significance was calculated by two-way ANOVA followed by Dunnett’s multiple comparisons test to compare LPS-treated cells versus untreated cells (**p < 0.01, ***p < 0.001) and followed by Tukey’s multiple comparisons test to compare KO and WT cells for each treatment condition (#*p* < 0.05, ###*p* < 0.001).

Transcriptomic data demonstrated the upregulation of *Nos2* and the downregulation of *Arg1* in response to LPS, especially in the KO cells, suggesting genotype-dependent modulation of NO secretion. Western blotting experiments confirmed the decreased expression of ARG1 in the *Abcd1^−/−^Abcd2^−/−^* cells in basal conditions (Figure 3A and Supplementary Figure S1 for densitometry analysis). The LPS treatment was shown to further increase NOS2 expression while downregulating ARG1, especially in the *Abcd1^−/−^Abcd2^−/−^* cells. We then analyzed NO production in the BV-2 cells upon LPS stimulation (Figure 3B). A significant increase in nitrite production was observed in the *Abcd1^−/−^Abcd2^−/−^* cells 7 h after LPS treatment, whereas an insignificant increase was observed in WT and *Acox1^−/−^* cells. Following a 24-h exposure to LPS, a notable elevation in NO production was detected in the KO cells but not in the WT cells. The increase was approximately 3-fold higher in the *Abcd1^−/−^Abcd2^−/−^* cells than in the *Acox1^−/−^* cells. Altogether, these findings confirm a disrupted redox balance in the peroxisomal mutant cells upon LPS activation, with *Abcd1^−/−^Abcd2^−/−^* cells displaying the most pronounced pro-oxidant status.

### Impact of BV-2 secretions on neurons and oligodendrocytes

We recently evidenced an increased secretion of DAM proteins, including cathepsins, in the mutant BV-2 cells in basal condition (Raas et al., 2023). We also demonstrated a pro-inflammatory skewing in the mutant cells upon LPS stimulation, resulting in excessive release of inflammatory cytokines (Tawbeh et al., 2023) coupled with an elevated production of oxidative stress markers. Here, we aimed to investigate the possible repercussions of these microglial alterations on neurons and oligodendrocytes. Microglial pathological state along with increased synthesis of ROS and NO has been postulated to intensify neuronal damage and accelerate neuronal death. This hypothesis is supported by a robust correlation observed in neurodegenerative diseases between immune activation and oxidative damage (Tönnies and Trushina, 2017; Simpson and Oliver, 2020). Therefore, we first investigated the viability of both neurons (mHypoA) and oligodendrocytes (158 N) in the presence of WT and mutant CM. We further analyzed neuronal morphology and function under these experimental conditions.

### Viability of neurons and oligodendrocytes

**Figure 4:**
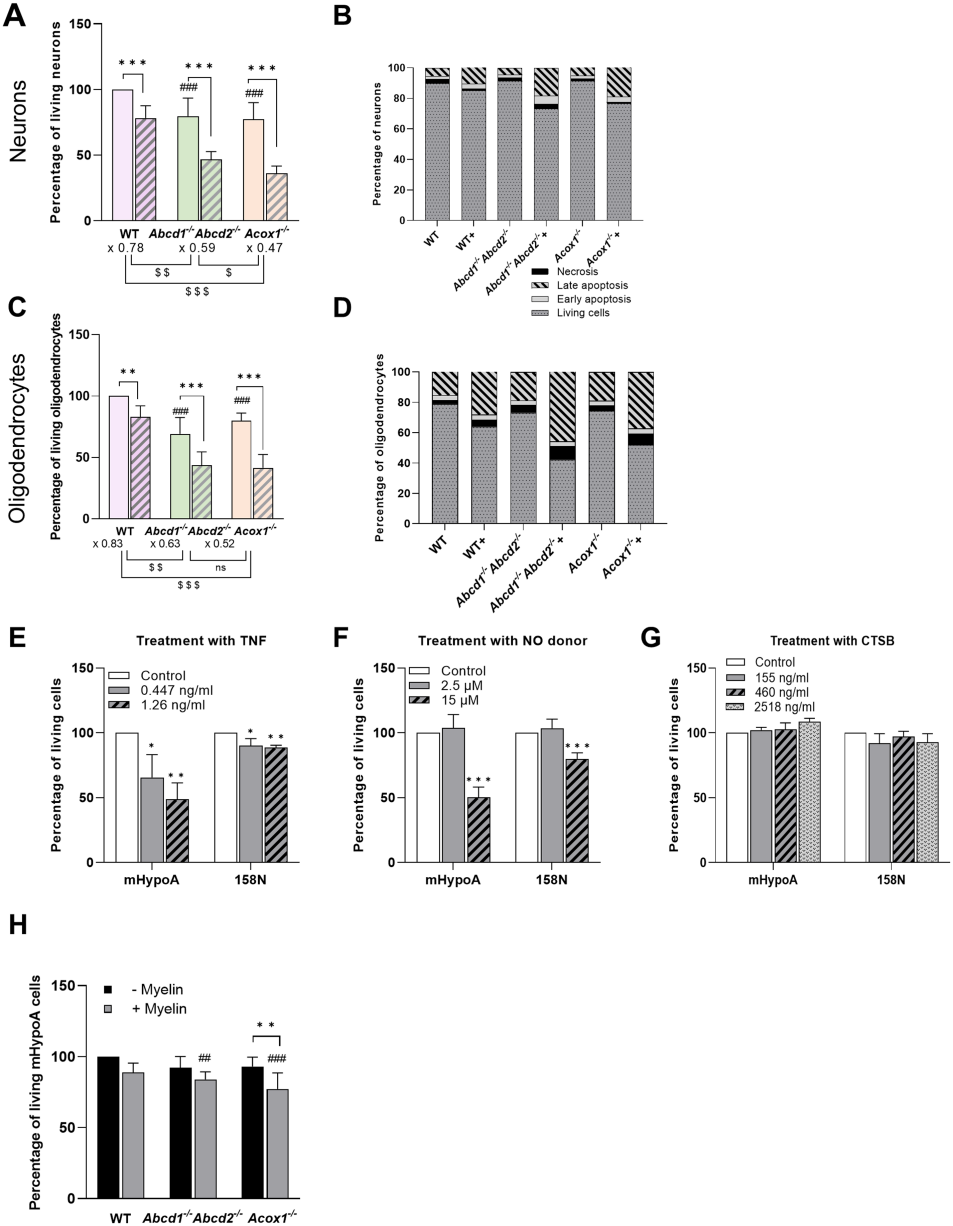
Impact of BV-2 conditioned media on the viability of mHypoA neurons and 158 N oligodendrocytes After being cultured for 48 h in the CM of WT and mutant BV-2 cells treated or not treated with LPS, the percentage + SD of viable neurons **(A)** and oligodendrocytes **(C)** was determined by MTT test. Statistical significance was calculated by 1-way ANOVA (uncorrected Fisher’s least significance difference). Comparison of cell viability between mutant and WT cells in the absence of LPS is indicated by ### (*p* < 0.001). Analysis of the LPS effect within each cell type is indicated by * (**p < 0.01; *** *p* < 0.001). “$” shows the comparison of the LPS effect between genotypes. The mode of cell death was identified by staining with annexin-V-fluorescein-isothiocyanate (FITC) and propidium iodide using an apoptosis detection kit followed by FACS. **(B,D)** Distribution of cells categorized as living (FITC^−^/IP^−^), early apoptotic (FITC^+^/IP^−^), late apoptotic (FITC^+^/IP^+^), or necrotic (FITC^−^/IP^+^) cells. After being cultured for 48 h in a medium supplemented with purified TNF **(E)**, NO donor **(F)**, and purified CTSB **(G)** at chosen concentrations corresponding to those previously measured in the secretome of the WT and mutant BV-2 cells (see Materials and Methods), the percentage of viable neurons was determined by MTT test. Data are shown as mean ± SD from 3 to 4 independent experiments. Data were analyzed within each cell type using one-way ANOVA followed by Dunnett’s multiple comparisons test (*p < 0.05; **p < 0.01; *** *p* < 0.001) to compare treated and untreated cells. **(H)** Cell viability of mHypoA cultured in CM of myelin-exposed BV-2 cells. After exposing WT and mutant BV-2 cells to myelin sheath debris for 24 h, their CM were used to culture mHypoA neurons. MTT test was used to investigate the cell viability at each condition. Data are shown as mean ± SD from three independent experiments and analyzed using two-way ANOVA followed by correction for false discovery rate (Tukey’s multiple comparisons test). “*” Is used to compare the myelin effect within the same cell type (**p < 0.01), whereas “#” is used to compare the myelin effect between mutant and WT cells (*^##^p* < 0.01; ^###^*p* < 0.001).

Using a classical cell viability MTT test, we assessed the percentage of viable cells under various conditions. Neurons or oligodendrocytes incubated in the CM of WT cells exhibited no significant difference compared with those incubated in fresh medium. Therefore, neurons or oligodendrocytes exposed to the CM of untreated WT BV-2 cells were designated as the control group. When mHypoA neurons were incubated in the CM of LPS-treated WT BV-2 cells, their viability decreased to 78% relative to the control (Figure 4A). Similarly, exposure to the CM of unstimulated *Abcd1^−/−^Abcd2^−/−^* cells resulted in a viability reduction to approximately 79%. Notably, the CM of LPS-treated *Abcd1^−/−^Abcd2^−/−^* cells significantly decreased neuronal viability to 47%. Similarly, incubation with the CM of *Acox1^−/−^* cells reduced neuronal viability to 77%, which further dropped to 36% upon LPS activation (Figure 4A). For 158 N oligodendrocytes, incubation with the CM of LPS-treated WT BV-2 cells decreased viability to 83% compared with the control. Exposure to the CM from *Abcd1^−/−^Abcd2^−/−^* cells reduced oligodendrocyte viability to 69%. This effect was further intensified when oligodendrocytes were exposed to CM from LPS-treated *Abcd1^−/−^Abcd2^−/−^* cells, decreasing viability to 44%. Similarly, CM from *Acox1^−/−^* cells decreased oligodendrocyte viability to 80%, which further dropped to 42% following LPS activation (Figure 4C).

Therefore, culturing neurons or oligodendrocytes in the CM of the mutant BV-2 cells significantly decreases cell viability, particularly under LPS activation. This finding strongly suggests that the CM derived from mutant BV-2 cells contains factors that are cytotoxic for neurons and oligodendrocytes. To determine whether the observed decrease in cell viability corresponds to apoptotic cell death, we used annexin-V-FITC/propidium iodide staining in combination with flow cytometric analysis. The results demonstrate that the cytotoxic effects induced by CM are predominantly associated with apoptotic cell death (Figures 4B,D). In the case of neurons treated with LPS-activated *Abcd1^−/−^Abcd2^−/−^* CM, the percentage of viable cells was 73.1%, while apoptotic and necrotic populations accounted for 23.7 and 3.1%, respectively. Similarly, oligodendrocytes exposed to LPS-treated *Abcd1^−/−^Abcd2^−/−^* CM displayed a decreased viable cell population (42.2%), with apoptotic and necrotic populations comprising 48.9 and 8.9%, respectively.

Such results indicate that the microglial secretome, especially that of mutant BV-2 cells upon LPS treatment, contains cytotoxic molecules capable of causing damage and death to CNS cells. Known microglia neurotoxins include cytokines such as TNF, enzymes such as cathepsins, amino acids such as glutamate, and small reactive molecules such as hydroxyl radicals and NO (Brown and Vilalta, 2015; Lindhout et al., 2021). To further explore the cause of the observed neurotoxicity, we subjected mHypoA and 158 N cells to specific concentrations of TNF, NO, and CTSB, previously measured in the secretome of the WT and KO BV-2 cells, treated or not treated with LPS.

We observed a substantial toxicity induced by TNF, with a dose-dependent effect on mHypoA. Specifically, at concentrations of 0.447 ng/mL reflecting the concentration measured in the supernatant of LPS-treated BV-2 WT cells, and 1.26 ng/mL corresponding to those of LPS-treated mutant BV-2 cells, cell viability dropped by 35 and 51%, respectively, compared with the control medium. While the impact on 158 N cells was somewhat lesser, there was still noticeable toxicity, with a decrease in viability of approximately 10% for both tested doses, still significantly lower than the control group (Figure 4E).

Neurons are highly sensitive to oxidative damage. As more NO is produced by LPS-treated mutant cells, we treated neurons and oligodendrocytes with SNAP, a NO donor, to replicate the NO concentrations observed in the secretome of LPS-treated mutant cells. Exposure to 50 μM SNAP resulted in a significant decrease in viability, with neurons experiencing a 53% reduction and oligodendrocytes showing a 22% reduction (15 μM). In contrast, no toxicity was detected at 6 μM SNAP (figure 4F).

Cathepsin B (CTSB), one of the DAM signature proteins, which are found to be upregulated in neurodegenerative diseases (Butovsky and Weiner, 2018), was found to induce lysosomal damage and neuronal death when released by exocytosis (Gabandé-Rodríguez et al., 2019). Here, we tested whether CTSB, which is oversecreted by the KO cells, induces cytotoxicity in neurons and oligodendrocytes. Our findings revealed no significant change in cell viability across the tested concentrations corresponding to the levels measured in the supernatants of WT, *Abcd1^−/−^Abcd2^−/−^* and *Acox1^−/−^* BV-2 cells, respectively (Raas et al., 2023) (Figure 4G).

Microglia respond to neural injury and neurodegeneration by undergoing pronounced morphological and cell biological transformations to clear myelin debris and promote repair (Santos and Fields, 2021). Such exposure to myelin debris has been shown to suppress or reduce microglial inflammatory activities *in vitro* in demyelinating disorders such as MS (Liu et al., 2006; Grajchen et al., 2020). As KO BV-2 cells demonstrated alterations in their phagocytic ability toward myelin (Tawbeh et al., 2023), we hypothesized that exposure of the KO BV-2 cells to myelin debris would modulate their expected anti-inflammatory response differently from that of WT cells. We, therefore, treated WT and KO BV-2 cells for 24 h with myelin debris and then collected their supernatants, which were subsequently used to culture mHypoA for 48 h. Viability assays indicate that the secretions from the myelin-exposed mutant cells still have a detrimental impact on neuronal viability, although the negative effects are not as pronounced as those observed with LPS treatment. There was a significant decrease in the number of neurons when incubated with the CM obtained from myelin-exposed *Abcd1^−/−^Abcd2^−/−^* and *Acox1^−/−^* cells compared with the controls (neurons incubated with WT CM). A significant decrease in neuronal viability was also observed with CM from myelin-exposed *Acox1^−/−^* cells than with CM of *Acox1^−/−^* cells (Figure 4H).

#### Morphology of neurons

**Figure 5:**
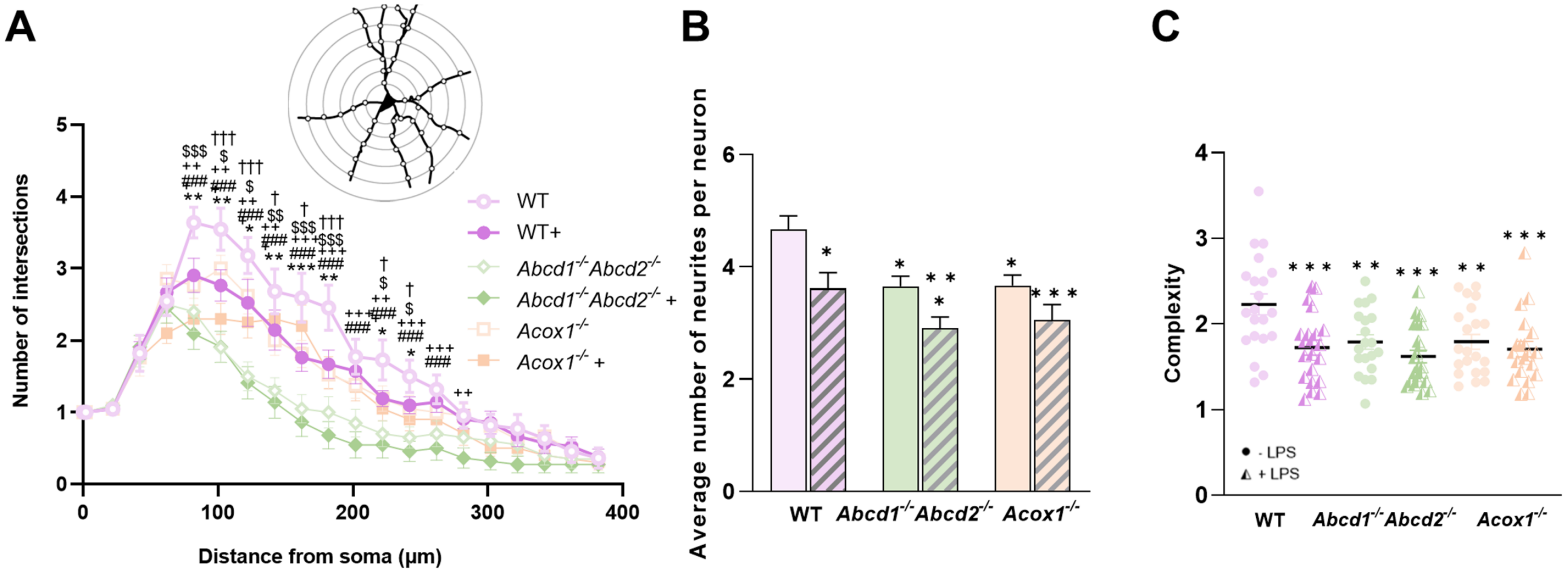
Impact of BV-2 conditioned media on the neuronal morphology. **(A)** Sholl profile of mHypoA cells incubated in the secretions of BV-2 cells for 48 h. Neurons seeded in CM from untreated WT BV-2 cells served as control. Statistical significance markers indicate comparison to the control. Symbols represent significance levels as follows: *p < 0.05, **p < 0.01 ***p < 0.001 for neurons cultured in LPS-stimulated WT cells CM; ###*p* < 0.001 for neurons cultured in untreated *Abcd1*^−/−^*Abcd2*^−/−^ cells CM; ++*p* < 0.01, +++*p* < 0.001 for neurons cultured in LPS-stimulated *Abcd1*^−/−^*Abcd2*^−/−^ cells CM; $*p* < 0.05, $$*p* < 0.01 $$$*p* < 0.001 for neurons cultured in untreated *Acox1*^−/−^ cells CM; and †*p* < 0.05, †††*p* < 0.001 for neurons cultured in LPS-stimulated *Acox1*^−/−^ cells CM. **(B)** Average number of neurites per neuron mHypoA cultured in CM of mutant and WT BV-2 treated or not treated with LPS. Statistical significance for the comparison to the CM from untreated WT BV-2 cells is indicated as *p < 0.05, **p < 0.01, ***p < 0.001. **(C)** Complexity of neurons was measured as the ratio of neurite–circle interactions to the number of circles intersecting each cell. Statistical significance for the comparison to the CM from untreated WT BV-2 cells is indicated as *p < 0.05, **p < 0.01, ***p < 0.001. Data are expressed as mean ± SEM (*n* = 18–20 cells from two independent experiments). The statistical differences were calculated using two-way ANOVA followed by correction for false discovery rate (Tukey’s multiple comparisons test).

The release of inflammatory molecules by activated microglia can lead to synaptic dysfunction, impair neuronal signaling pathways, and promote neuronal death. These processes can ultimately result in a reduction in neuronal complexity, which refers to the structural and functional intricacy of neuronal connections and processes (Muzio et al., 2021). To assess whether the morphological parameters of neurons were also affected by the CM derived from the mutant BV-2 cells, we performed Sholl analysis, a classical morphological test, on neurons subjected to different CM conditions. The Sholl intersection profile displayed significant differences among the different conditions with a decreased number of intersections in neurons cultured in CM of mutant cells, especially upon LPS treatment (Figure 5A). Two-way ANOVA analysis of the number of neurite branches in each neuron revealed a significant effect of LPS treatment, but a more pronounced impact was observed within the mutant BV-2 cells CM (Figure 5B) The average number of neurites per neuron decreased from 4.6 in the presence of CM from WT cells to 3.6 with LPS-treated WT CM. Notably, the number of neurites decreased from 3.6 with *Abcd1^−/−^Abcd2^−/−^* CM to 2.9 with LPS-treated *Abcd1^−/−^Abcd2^−/−^* CM. In the case of neurons incubated with *Acox1^−/−^* CM, the number of neurites decreased from 3.6 to 3 upon LPS treatment. Further analysis was performed to assess neuronal complexity under each condition. Neuronal complexity was calculated by dividing the sum of circles-neurites crossovers by the number of circles intersecting a cell. Notably, neuronal complexity was significantly decreased when neurons were incubated with mutant BV-2 CM than with WT BV-2 CM (Figure 5C). However, LPS treatment did not result in significant differences when comparing the CM of LPS-treated WT and mutant cells.

#### Function of neurons

**Figure 6:**
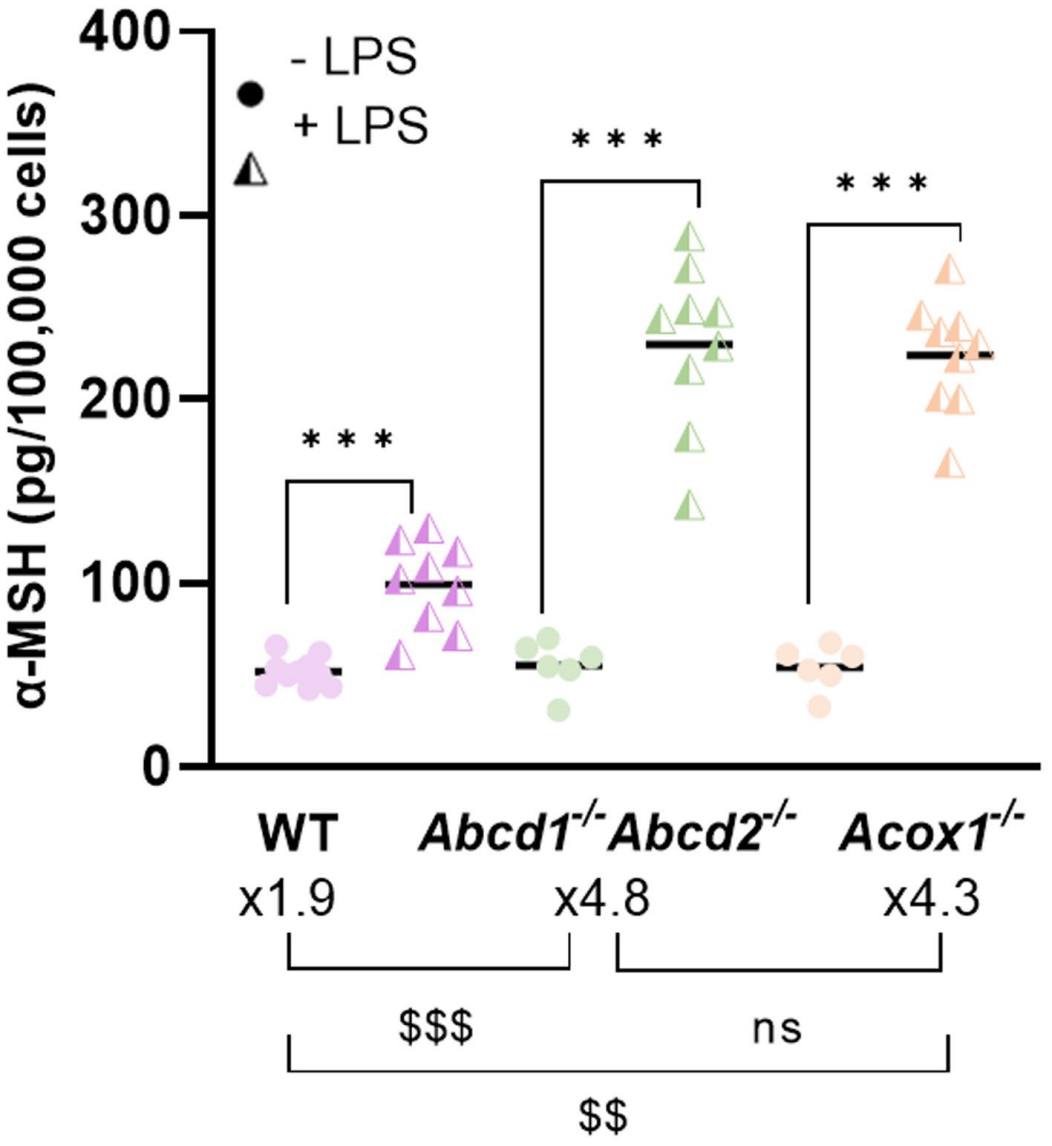
Impact of BV-2 conditioned media on *α*-MSH secretion by neurons. α-MSH secretion by mHypoA neurons after 48 h incubation in BV-2 WT, *Abcd1^−/−^Abcd2^−/−^*, and *Acox^−/−^* CM treated or not treated with LPS. Data were normalized over the number of cells and expressed as pg./100,000 cells. * Represents statistical significance for the comparison of the LPS effect within a genotype. $ Represents statistical significance for the LPS effect between the genotypes with *p* < 0.01; $$$ for *p* < 0.001. The statistical differences were calculated using two-way ANOVA followed by correction for false discovery rate (Tukey’s multiple comparisons test) from three technical and three biological replicates.

Regarding the changes in morphology, we considered it crucial to further explore the hypothesis that a peroxisomal defect in microglia may not only elicit cytotoxic effects but also alter neuronal functionality. In the brain, a neuroinflammatory state can deeply modify the activity of neurons and circuits. For instance, in the hypothalamus, a brain region that controls food intake, TNF*α* inhibits orexigenic AgRP neurons and activates anorexigenic proopiomelanocortin (POMC) neurons, promoting inflammation-associated anorexia. In the context of control of feeding behavior, TNF was found to inhibit the activity of AgRP-producing neurons and depolarize proopiomelanocortin (POMC) neurons (Chaves et al., 2020). When secreted by POMC neurons, the alpha-melanocyte-stimulating hormone (α-MSH), which derives from the POMC propeptide, reduces food intake (Cone, 2006). Here, we investigated the secretion of α-MSH upon stimulation by BV-2 CM in the POMC-expressing mHypoA cell line (Belsham et al., 2004; Nazarians-Armavil et al., 2014). Incubation of mHypoA neurons with the CM of WT BV-2 cells resulted in α-MSH production of 52 pg./100,000 cells, a result not significantly different from that obtained with CM of mutant BV-2 cells. However, when exposed to the CM from LPS-activated WT BV-2 cells, the α-MSH production increased to 99 pg./100,000 cells. Notably, the induction of α-MSH secretion was more pronounced when mHypoA neurons were incubated with secretions from LPS-activated BV-2 KO cells and drastically increased to 230 and 223 pg./100,000 cells when the CM were derived from LPS-activated *Abcd1^−/−^Abcd2^−/−^* cells or *Acox1^−/−^* cells, respectively (Figure 6).

## Discussion

Our study aimed to explore the effects of peroxisomal impairment in microglia and their possible consequences on neighboring oligodendrocytes and neurons. We previously evidenced various cellular alterations in BV-2 cells, including reprogramming of lipid metabolism and modification of lysosomal and autophagic functions, as well as the induction of a pathological state characterized by the secretion of various DAM proteins (Raas et al., 2023). In addition, we revealed an amplified response to LPS with an exaggerated inflammatory response and modulation of immune functions such as phagocytosis and antigen presentation by microglia (Tawbeh et al., 2023). To deepen our understanding, we reanalyzed the transcriptomic data focusing on genes involved in oxidoreduction processes and demonstrated a moderated change in the expression pattern of several important genes affecting both the production of ROS and RNS and the antioxidant defense. These modifications, although not significantly altering the basal state of microglia, could appear as a priming event rendering cells hypersensitive to a secondary stimulus, as described in neurodegenerative diseases such as Alzheimer’s disease (Perry and Holmes, 2014; Lima et al., 2022). Accordingly, when exposed to LPS and as in the case of the inflammatory response observed in mutant BV-2 cells (Tawbeh et al., 2023), the mutant cell lines demonstrated a more pronounced response both in the number of redox-related DEGs and in their fold change than in the LPS-treated WT cells. *Nrf2* and *Keap1* are the main regulators of redox status, especially in microglia (Rojo et al., 2014), but we did not observe significant modifications of their expression in the mutant genotypes, either in basal conditions or upon LPS activation. However, key genes encoding ROS-producing enzymes such as *Cybb* (NOX2) were upregulated in LPS-treated WT and mutant cells, with significant upregulation in the *Acox1*^−/−^ cells than in the WT cells. The activation of NOX represents the main source of ROS in microglia (Block and Hong, 2005; Simpson and Oliver, 2020) and has been implicated in the pathogenesis of experimental autoimmune encephalomyelitis (EAE). *Cybb* ablation was indeed shown to inhibit ROS production in microglia and attenuate neuroinflammation (Hu et al., 2021).

Furthermore, we showed here that the expression of genes involved in antioxidant defense was increased and confirmed this observation at the protein level for GSR, PRDX5, and CAT. Genes involved in nitric oxide homeostasis (*Arg1* and *Nos2*) were also in the hits of the RNA-seq analysis, and we confirmed at the protein level the genotype differences and the exaggerated response to LPS. This overactivation resulted in a large increase in ROS and NO secretion than in the controls. In microglial cells, ROS have been shown to activate the NF-kappaB pathway, which controls the secretion of pro-inflammatory cytokines (Park et al., 2015). NO is also known as a regulator of inflammation and immune function of microglia such as phagocytosis (Scheiblich and Bicker, 2016). Therefore, the previously documented alterations of the phagocytosis ability of mutant BV-2 cells (Tawbeh et al., 2023) could be linked to the increase in NO production. Altogether, our findings in the mutant cells show that both ROS and NO production, and pro-inflammatory cytokine secretion, are heightened in response to LPS than in WT cells. Whether the alteration of redox homeostasis overlaps or causes the recruitment of microglia in an exaggerated pro-inflammatory state *in vivo* remains to be demonstrated.

The phenotypic heterogeneity of X-ALD remains enigmatic. However, it is obvious that dysfunction in microglia and the presence of oxidative stress play significant roles in the physiopathology of both cerebral demyelinating forms and adrenomyeloneuropathy. Oxidative stress, a common feature in neurodegenerative diseases, has been proposed to be the initial hit in the pathogenesis of X-ALD, settling the inflammatory cascade and causing cell death (Singh and Pujol, 2010). Differential responses to both internal dysfunction and external environmental factors may explain the onset and progression of clinical manifestations. Furthermore, excess of ROS in glia has been shown as the major contributor to the demyelination in patients with Mitchell syndrome caused by a gain of function mutation in the *ACOX1* gene (Chung et al., 2020; Raas et al., 2024). In this study and previously, we showed that microglial cells with peroxisomal defects are affected at various levels, which in turn impact their ability to respond to LPS activation. Microglia with their cellular contacts and secretions are indeed known to communicate with all cerebral cell types, modulate the integrity of the blood–brain barrier and ensure the recruitment and activation of peripheral cells (Wright-Jin and Gutmann, 2019; Mayer and Fischer, 2024). The overproduction of ROS, NO, inflammatory cytokines, and secretory DAM proteins likely has consequences on various brain cells. Therefore, we decided to explore the paracrine effects of microglial secretion. Our study was focused on two neuron and oligodendrocyte cell lines, and these investigations were conducted under both basal conditions and in the presence of LPS as a secondary stimulus. Of note, the effects of cell-to-cell contacts were not studied here but definitely deserve investigation (Szepesi et al., 2018). We indeed demonstrated several important modifications affecting the membranes of the mutant BV-2 cells (lipid content, expression of membrane proteins involved in adhesion and signaling) and observed a massive downregulation of the expression of Cx3cr1 in the mutant BV-2 cells (Raas et al., 2023; Tawbeh et al., 2023). Of note, the CX3CR1/CX3CL1 axis is one of the main communication channels between microglia and neurons (Pawelec et al., 2020).

CM of LPS-activated primary microglia or BV-2 cells have been shown to produce apoptosis of different cell types including retinal pericytes (Ding et al., 2017), pheochromocytoma PC12 cells (Dai et al., 2015; Kaewmool et al., 2020), or neural precursor cells and primary cortical mouse neurons (Kim et al., 2007; Guadagno et al., 2015). Here, using the CM from our LPS-activated WT and mutant BV-2 cells, we demonstrated a cytotoxic effect on both mHypoA neurons and 158 N oligodendrocytes, primarily through apoptotic cell death. Apoptosis was significantly increased using CM from KO cells, suggesting that microglia with peroxisomal defects promote a further toxic environment and that specific factors released by the mutant BV-2 cells are either absent or at a reduced level in the CM of WT cells upon LPS activation. ROS that are transiently overproduced by mutant BV-2 cells could diffuse through the plasma membrane or be brought by extracellular vesicles, thus contributing to this cytotoxicity (Vilhardt et al., 2017). The intracellular signaling effects of ROS in microglia may also have contributed indirectly by inducing the secretion of inflammatory cytokines in excess. TNF produced by LPS-activated microglia was found necessary and sufficient to trigger apoptosis in neural precursor cells (Guadagno et al., 2015). Previous research has demonstrated the involvement of microglial CTSB in driving neurodegenerative diseases primarily through neuroinflammation induction (Nakanishi, 2020; Pislar et al., 2021). NO release, which appears to be hugely increased in LPS-activated mutant cells, could also be responsible for the toxic effects. Our tests using isolated TNF, CTSB, and NO donors suggest that TNF and NO, but not CTSB, contribute to the apoptotic signaling pathways on mHypoA neurons and 158 N oligodendrocytes. As peroxisomal defects affect phagocytosis ability of BV-2 cells (Tawbeh et al., 2023) and phagocytosis activity is intimately linked to inflammatory control (Liu et al., 2006; Grajchen et al., 2020), we also tested the CM of BV-2 cells exposed to myelin debris. Although we expected reduced toxicity, surprisingly, CM from cells exposed to myelin revealed greater toxicity, regardless of the genotype. CM from *Acox1*^−/−^ cells, which display the highest phagocytosis activity, induced apoptosis even more efficiently. A possible explanation would be that the KO cells present several autophagic and lysosomal alterations and are already overwhelmed with the accumulated VLCFA (Raas et al., 2023). Engulfing more lipids would further stress the cells, thus rendering them more pro-inflammatory. It is important to note that these results represent an initial exploration into understanding the cytotoxic factors secreted by microglia with peroxisomal defects. Comprehensive analyses are essential to fully unravel the complexity of this phenomenon and its underlying mechanisms. Many other secreted molecules such as cathepsin D, other pro-inflammatory cytokines, or prostaglandins may also contribute to the evidenced toxicity. Proteomic studies have detected 187 and 155 proteins in supernatants of murine BV-2 and primary microglia, respectively, and demonstrated a significant difference in secretomes between unstimulated and immune-stimulated cells. A much larger number of proteins (4938) has been detected in supernatants of IFN-*γ* or LPS-stimulated BV-2 cells (Woo et al., 2017).

Beyond toxicity, we were interested in studying the impact of microglial secretion on the morphology and function of neurons. Sholl profile of mHypoA neurons indicated a decreased ramification of neurons, especially those incubated with *Abcd1^−/−^Abcd2^−/−^* CM. The reduction in the number of neurites and the overall decrease in neuronal complexity suggest impaired neurite outgrowth and ramifications. These alterations in neuronal morphology may have implications for neuronal connectivity and proper functioning and are a characteristic of neurodegenerative diseases (Kovacs, 2019). The underlying cause of the observed alterations in neuronal morphology could be attributed to the reduced trophic support, as the absence of proper peroxisomal functioning may alter a wide range of microglial functions and lead to reduced secretion of neurotrophic factors such as neurotrophin-3, brain-derived neurotrophic factor, or even TGF-*β*, thereby impacting neurite outgrowth. The excessive inflammation induced by LPS treatment and demonstrated by increased secretion of pro-inflammatory cytokines, as well as the increased production of NO and ROS, can be additional important factors. Neurite outgrowth of human neurons was shown to be inhibited by NO, and the use of an NOS blocker reversed this inhibition (Scheiblich and Bicker, 2016). Further investigations are warranted to elucidate the underlying mechanisms through which CM from mutant microglia modulate neuronal morphology, first by using different neuronal cell types and then by investigating the cytoskeletal rearrangements in the neurons including the assembly of cytoskeletal proteins forming neurite extensions and the expression of related genes. Regarding the function of neurons, our study based on the use of the mHypoA neuron cell line was only limited to the secretion of the *α*-MSH neuropeptide. We clearly demonstrated a more pronounced secretion of α-MSH when using CM from LPS-activated mutant BV-2 cells indicating that a factor, present in the secretome of the LPS-activated mutant BV-2 cells, is associated with the more pronounced activation of neurons. Several stimuli can trigger α-MSH processing and secretion (Wu et al., 2023). Given that the main function of peroxisomes is related to lipid metabolism and that α-MSH secretion occurs on a high-fat diet, it is tempting to propose that the peroxisomal defect, which is associated with VLCFA accumulation and mimics a situation of lipid overload, could trigger a microglial signal to neurons, possibly a pro-inflammatory signal. Interestingly, α-MSH has been shown to inhibit NO and TNF production by microglia (Wu et al., 2023) suggesting a crosstalk between microglia and neurons in which peroxisomes may play a pivotal role.

From a pathological point of view, these *in cellulo* data provide an initial glimpse and may not fully capture the main features of the pathophysiology of peroxisomal diseases, especially X-ALD, which is a very complex disorder presenting variability both in the onset and the neurological symptoms (Trompier and Savary, 2013; Yska et al., 2024). However, they offer valuable insights. Although hypothalamic neurons are not a primary focus in the cerebral form of X-ALD, whose pathogenesis involves a global inflammatory demyelination starting in the corpus callosum, our findings contribute to understand how peroxisomal defect affects microglia and its abilities to communicate with other brain cells. In X-ALD, microglial defects and axonal damages are likely early events that may mutually reinforce each other, leading to demyelination. A major microglial dysfunction combined with the rupture of the blood–brain barrier likely amplifies inflammation and contributes to the spread of microglial defects, demyelination, and nervous lesions (Yska et al., 2024). In this story, ROS, NO, and inflammatory cytokines originating from microglia appear to be key pathogenic components, and the characterization of the mutant microglial cells as well as the effect of their secretion on neurons and oligodendrocytes align with this hypothesis. Our previous results (Raas et al., 2023; Tawbeh et al., 2023) and the present data confirm a pathological state and an overreactivity to LPS stimulation resulting in increased secretion of various signaling molecules (TNF, DAM proteins, ROS, and NO). We documented how peroxisomal defects in microglia can result in paracrine effects on surrounding neurons and oligodendrocytes leading to apoptosis or affecting cellular functions. A recent study demonstrated that the intranasal delivery of BV-2-derived extracellular vesicles affects the inflammatory profile of microglia and triggers cognitive modifications in mice (Rinaldi et al., 2024). Secretions from brain microglia presenting peroxisomal defects may likely diffuse extensively to the rest of the brain cells, contributing to the spread of pathological lesions. Therefore, for therapeutic purposes, targeting microglia in order to correct or attenuate their initial defect and its consequences in terms of activation and secretion of signaling molecules and toxicity makes perfect sense. Beyond peroxisomal leukodystrophies, common neurodegenerative and age-related diseases such as multiple sclerosis or Alzheimer’s disease show similarities in microglial defects and pathogenic events. *In vitro* studies and clinical observations suggest that peroxisomal dysfunction may also play a role in these diseases (Jo and Cho, 2019; Zarrouk et al., 2020). Further studies and increased attention on peroxisomes, an often overlooked organelle, in common neurodegenerative disorders would be beneficial.

In conclusion, our study underscores the critical role of peroxisomal integrity in maintaining microglial homeostasis. Peroxisome defects in microglia contribute to abnormal activation and secretion profiles, driving neuroinflammation and cellular dysfunction in surrounding neurons and oligodendrocytes. It reinforces the relevance of microglia as a therapeutic target in peroxisomal and neurodegenerative diseases.

## Data Availability

The datasets presented in this study can be found in online repositories. The names of the repository/repositories and accession number(s) can be found at: https://www.ncbi.nlm.nih.gov/, GSE200022; https://www.ncbi.nlm.nih.gov/, GSE237635.

## Footnotes

^1^https://www.ncbi.nlm.nih.gov/geo/query/acc.cgi?acc=GSE200022

^2^https://bioinfogp.cnb.csic.es/tools/venny/index.html

## Supplementary material

SUPPLEMENTARY FIGURE 1 Densitometric analysis of western blotting experiments Bar graphs represent the densitometric analysis of immunoblots of proteins related to oxidative stress (NOX1, GSR, PDRX5, CAT) and NO production (NOS2, ARG1) in *Abcd1*^-/-^ *Abcd2*^-/-^, *Acox1*^-/-^ and WT BV-2 cells treated or not with LPS for 7 h or 24 h. Each bar corresponds to the mean of 3 independent experiments +SEM. Statistical significance was calculated by Anova 2-ways analysis followed by Tukey’s multiple comparisons test (**p* < 0.05; ***p* < 0.01; ****p* < 0.001 for comparison of LPS-treated cells versus untreated cells and #*p* < 0.05; ##*p* < 0.01 for comparison of KO cells versus WT cells). Source data are available online for this figure.

SUPPLEMENTARY PRESENTATION 1 Uncropped western blots source data related to Figure 2E and Figure 3A.
