# Supplementary figures and images for: Impaired peroxisomal beta-oxidation in microglia triggers oxidative stress and impacts neurons and oligodendrocytes
Source: Front Mol Neurosci. 2025 Jan 30;18:1542938. doi: 10.3389/fnmol.2025.1542938 (PMC11826809; doi:10.3389/fnmol.2025.1542938)

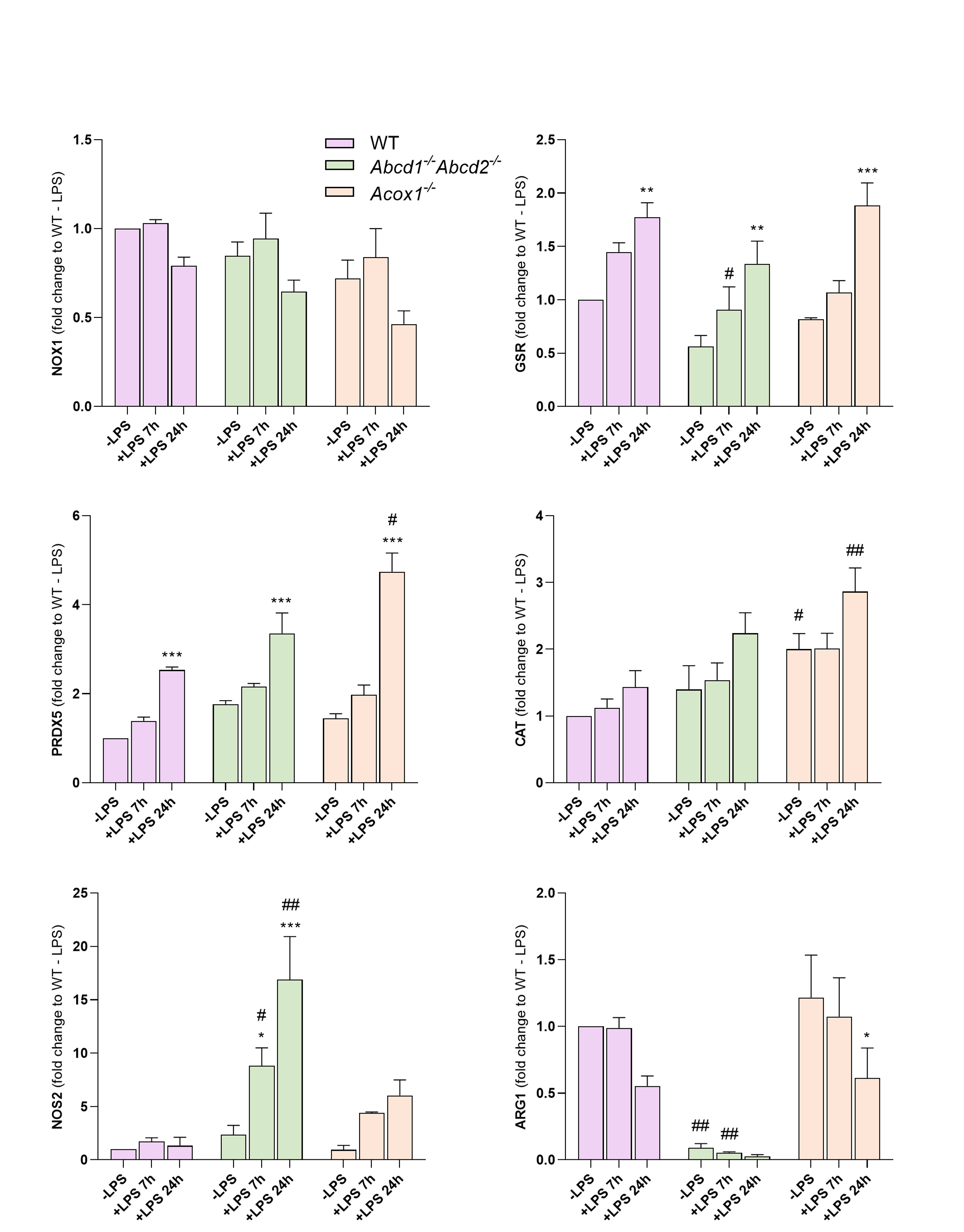

Supplement: SUPPLEMENTARY FIGURE 1 — Densitometric analysis of western blotting experiments Bar graphs represent the densitometric analysis of immunoblots of proteins related to oxidative stress (NOX1, GSR, PDRX5, CAT) and NO production (NOS2, ARG1) in Abcd1-/- Abcd2-/-, Acox1-/- and WT BV-2 cells treated or not with LPS for 7 h or 24 h. Each bar corresponds to the mean of 3 independent experiments +SEM. Statistical significance was calculated by Anova 2-ways analysis followed by Tukey’s multiple comparisons test (*p < 0.05; **p < 0.01; ***p < 0.001 for comparison of LPS-treated cells versus untreated cells and #p < 0.05; ##p < 0.01 for comparison of KO cells versus WT cells). Source data are available online for this figure. [file Image_1.TIF]
