# Supplementary material for: Impaired peroxisomal beta-oxidation in microglia triggers oxidative stress and impacts neurons and oligodendrocytes
Source: Front Mol Neurosci. 2025 Jan 30;18:1542938. doi: 10.3389/fnmol.2025.1542938 (PMC11826809; doi:10.3389/fnmol.2025.1542938)
Supplement: SUPPLEMENTARY PRESENTATION 1 — Uncropped western blots source data related to Figure 2E and Figure 3A. [file Presentation_1.PPTX]

## Slide 1
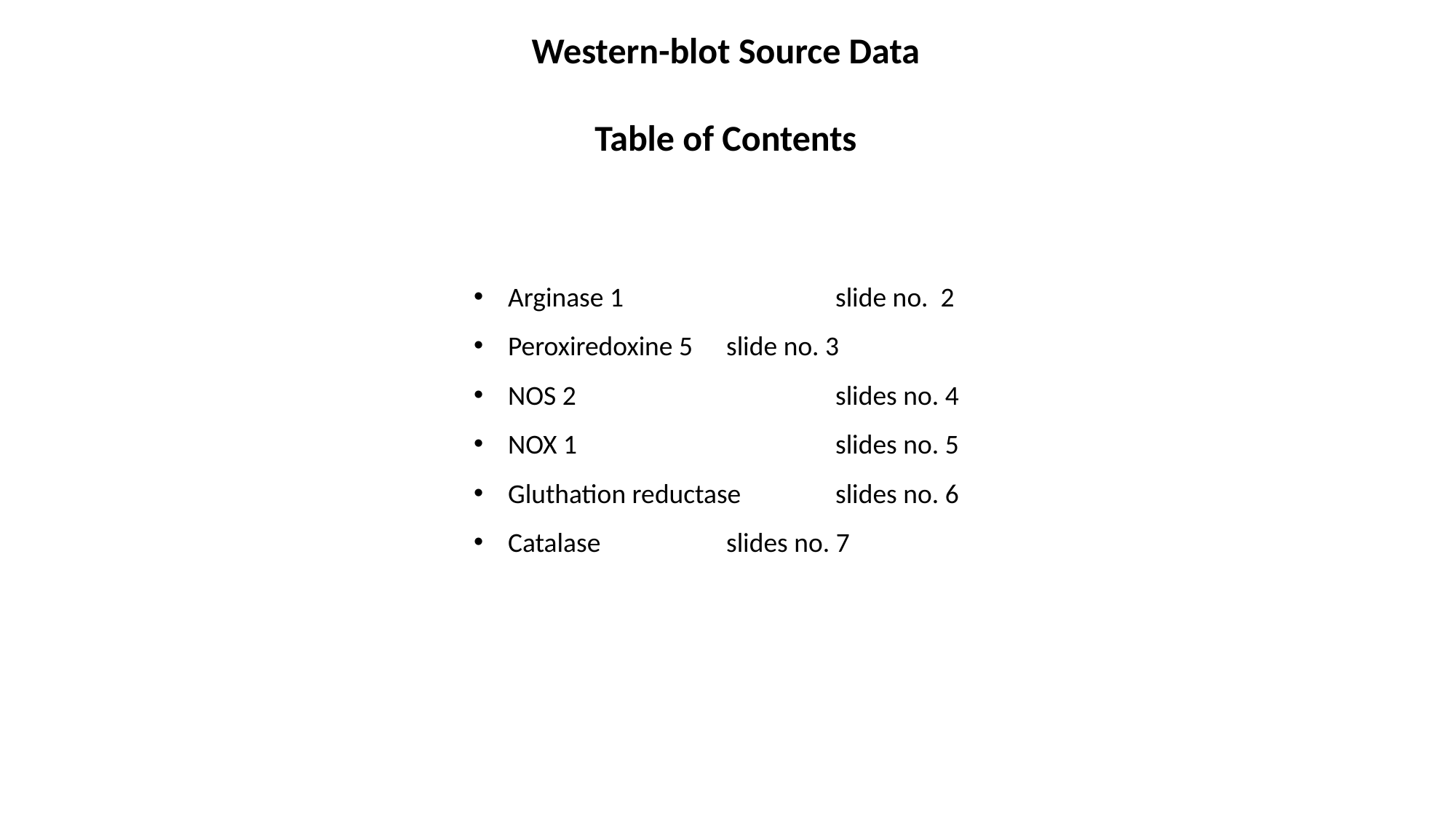

Western-blot Source Data
Table of Contents
Arginase 1		slide no. 2
Peroxiredoxine 5	slide no. 3
NOS 2			slides no. 4
NOX 1		 	slides no. 5
Gluthation reductase	slides no. 6
Catalase		slides no. 7

## Slide 2
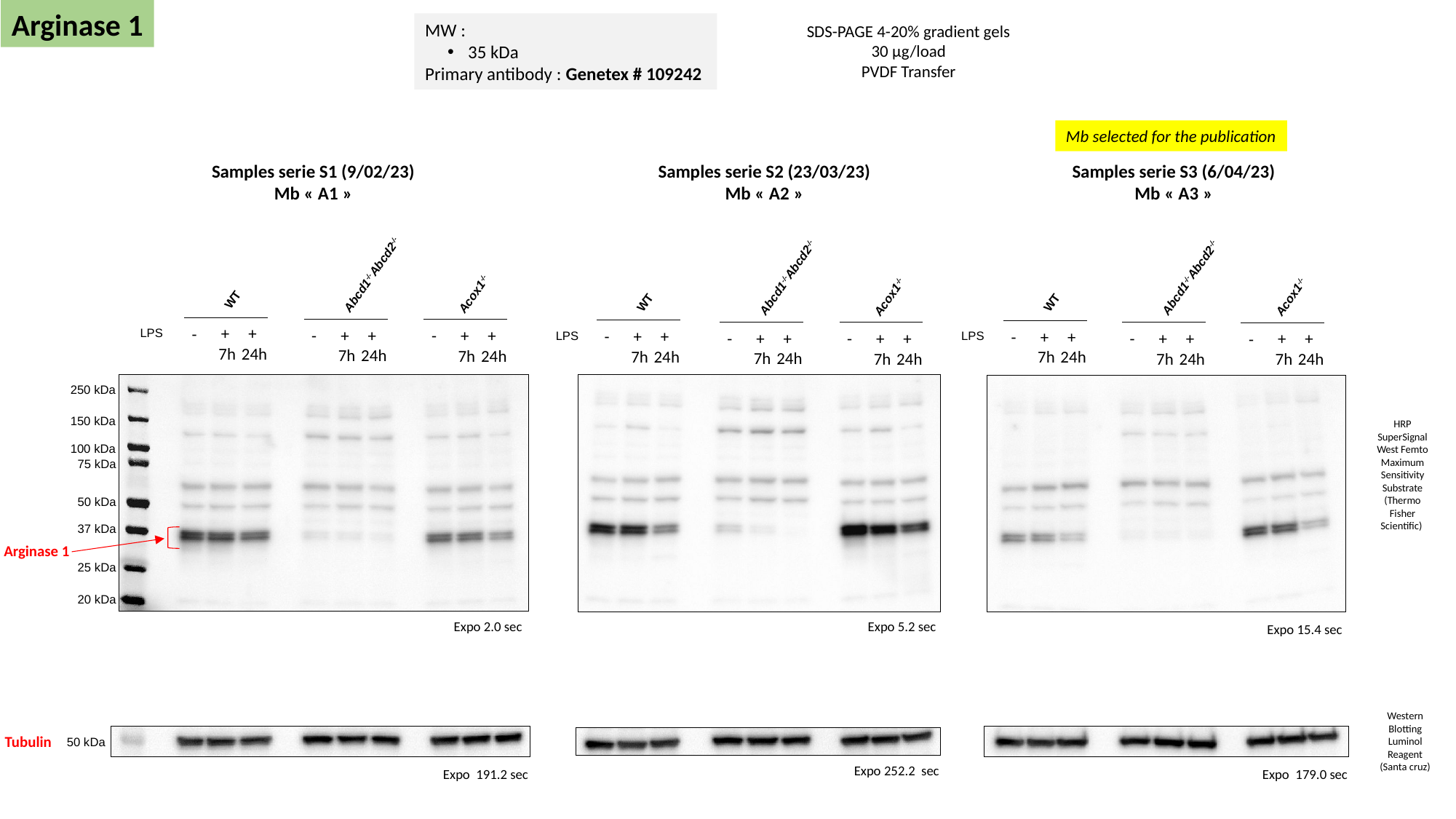

Arginase 1
MW :
35 kDa
Primary antibody : Genetex # 109242
SDS-PAGE 4-20% gradient gels
30 µg/load
PVDF Transfer
Mb selected for the publication
Samples serie S1 (9/02/23)
Mb « A1 »
Samples serie S2 (23/03/23)
Mb « A2 »
Samples serie S3 (6/04/23)
Mb « A3 »
Abcd1-/-Abcd2-/-
-
+
7h
+
24h
Abcd1-/-Abcd2-/-
-
+
7h
+
24h
Abcd1-/-Abcd2-/-
-
+
7h
+
24h
Acox1-/-
Acox1-/-
Acox1-/-
WT
WT
WT
-
+
7h
+
24h
-
+
7h
+
24h
LPS
-
+
7h
+
24h
-
+
7h
+
24h
-
+
7h
+
24h
-
+
7h
+
24h
LPS
LPS
250 kDa
150 kDa
100 kDa
75 kDa
50 kDa
37 kDa
25 kDa
20 kDa
HRP SuperSignal West Femto Maximum Sensitivity Substrate (Thermo Fisher Scientific)
Arginase 1
Expo 2.0 sec
Expo 5.2 sec
Expo 15.4 sec
Western Blotting Luminol Reagent (Santa cruz)
Tubulin
50 kDa
Expo 252.2  sec
Expo  191.2 sec
Expo  179.0 sec

## Slide 3
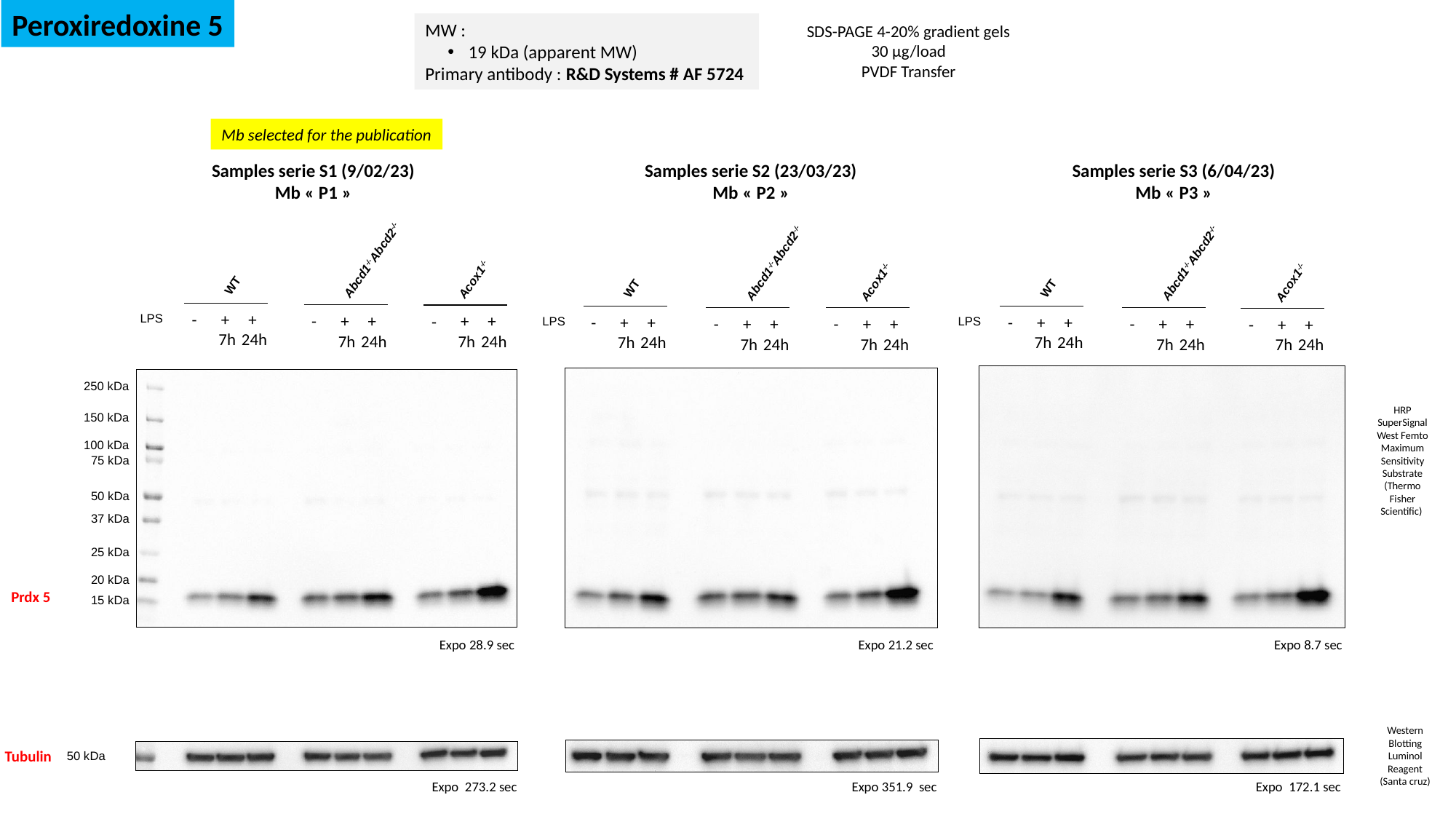

Peroxiredoxine 5
MW :
19 kDa (apparent MW)
Primary antibody : R&D Systems # AF 5724
SDS-PAGE 4-20% gradient gels
30 µg/load
PVDF Transfer
Mb selected for the publication
Samples serie S1 (9/02/23)
Mb « P1 »
Samples serie S2 (23/03/23)
Mb « P2 »
Samples serie S3 (6/04/23)
Mb « P3 »
Abcd1-/-Abcd2-/-
-
+
7h
+
24h
Abcd1-/-Abcd2-/-
-
+
7h
+
24h
Abcd1-/-Abcd2-/-
-
+
7h
+
24h
Acox1-/-
Acox1-/-
Acox1-/-
WT
WT
WT
-
+
7h
+
24h
-
+
7h
+
24h
LPS
-
+
7h
+
24h
-
+
7h
+
24h
-
+
7h
+
24h
-
+
7h
+
24h
LPS
LPS
250 kDa
150 kDa
100 kDa
75 kDa
50 kDa
37 kDa
25 kDa
20 kDa
HRP SuperSignal West Femto Maximum Sensitivity Substrate (Thermo Fisher Scientific)
Prdx 5
15 kDa
Expo 28.9 sec
Expo 21.2 sec
Expo 8.7 sec
Western Blotting Luminol Reagent (Santa cruz)
Tubulin
50 kDa
Expo  273.2 sec
Expo 351.9  sec
Expo  172.1 sec

## Slide 4
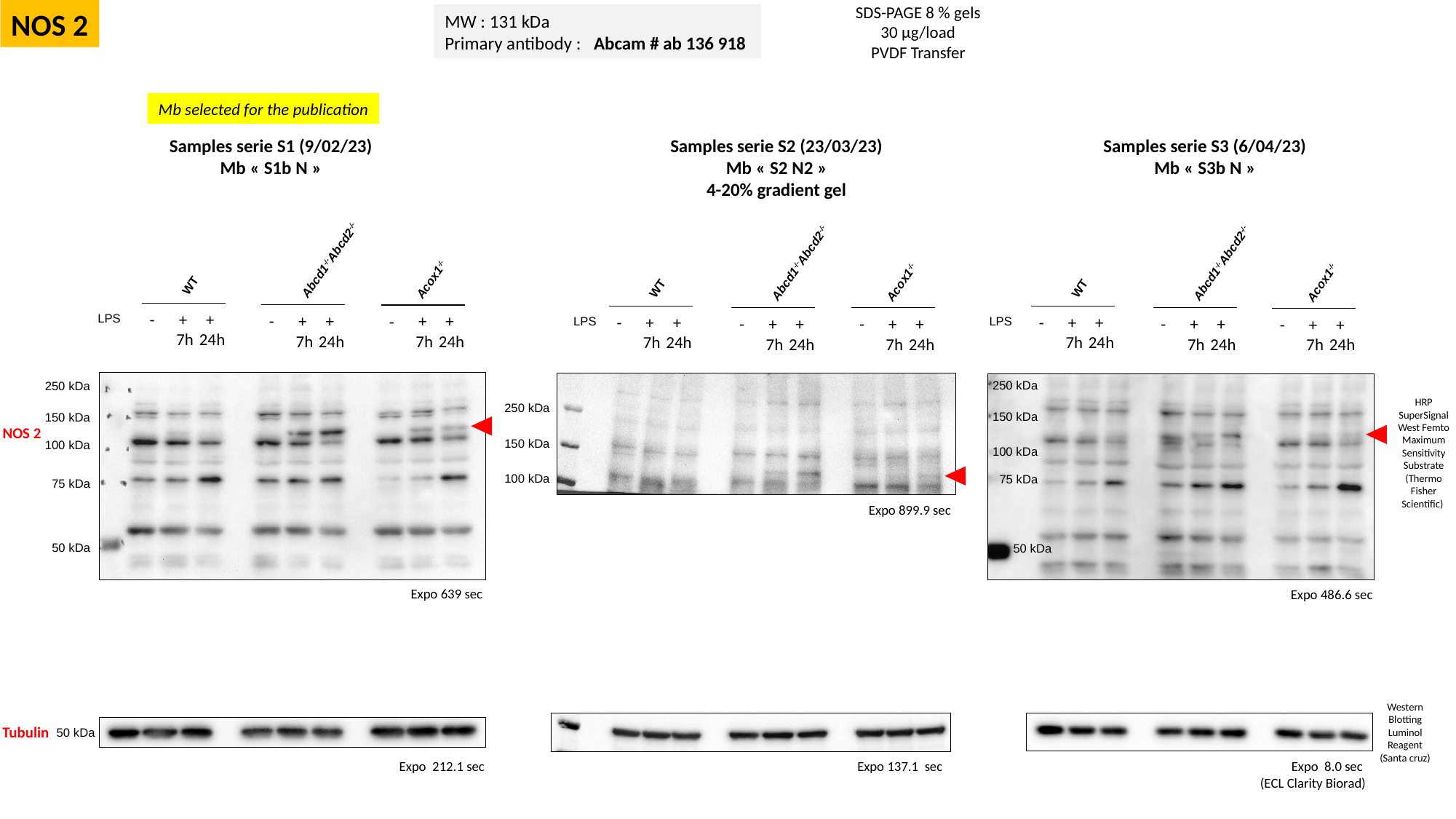

NOS 2
SDS-PAGE 8 % gels
30 µg/load
PVDF Transfer
MW : 131 kDa
Primary antibody :   Abcam # ab 136 918
Mb selected for the publication
Samples serie S1 (9/02/23)
Mb « S1b N »
Samples serie S2 (23/03/23)
Mb « S2 N2 »
4-20% gradient gel
Samples serie S3 (6/04/23)
Mb « S3b N »
Abcd1-/-Abcd2-/-
-
+
7h
+
24h
Abcd1-/-Abcd2-/-
-
+
7h
+
24h
Abcd1-/-Abcd2-/-
-
+
7h
+
24h
Acox1-/-
Acox1-/-
Acox1-/-
WT
WT
WT
-
+
7h
+
24h
-
+
7h
+
24h
LPS
-
+
7h
+
24h
-
+
7h
+
24h
-
+
7h
+
24h
-
+
7h
+
24h
LPS
LPS
250 kDa
150 kDa
100 kDa
75 kDa
50 kDa
250 kDa
150 kDa
100 kDa
75 kDa
50 kDa
HRP SuperSignal West Femto Maximum Sensitivity Substrate (Thermo Fisher Scientific)
250 kDa
NOS 2
150 kDa
100 kDa
Expo 899.9 sec
Expo 639 sec
Expo 486.6 sec
Western Blotting Luminol Reagent (Santa cruz)
Tubulin
50 kDa
Expo  212.1 sec
Expo 137.1  sec
Expo  8.0 sec
(ECL Clarity Biorad)

## Slide 5
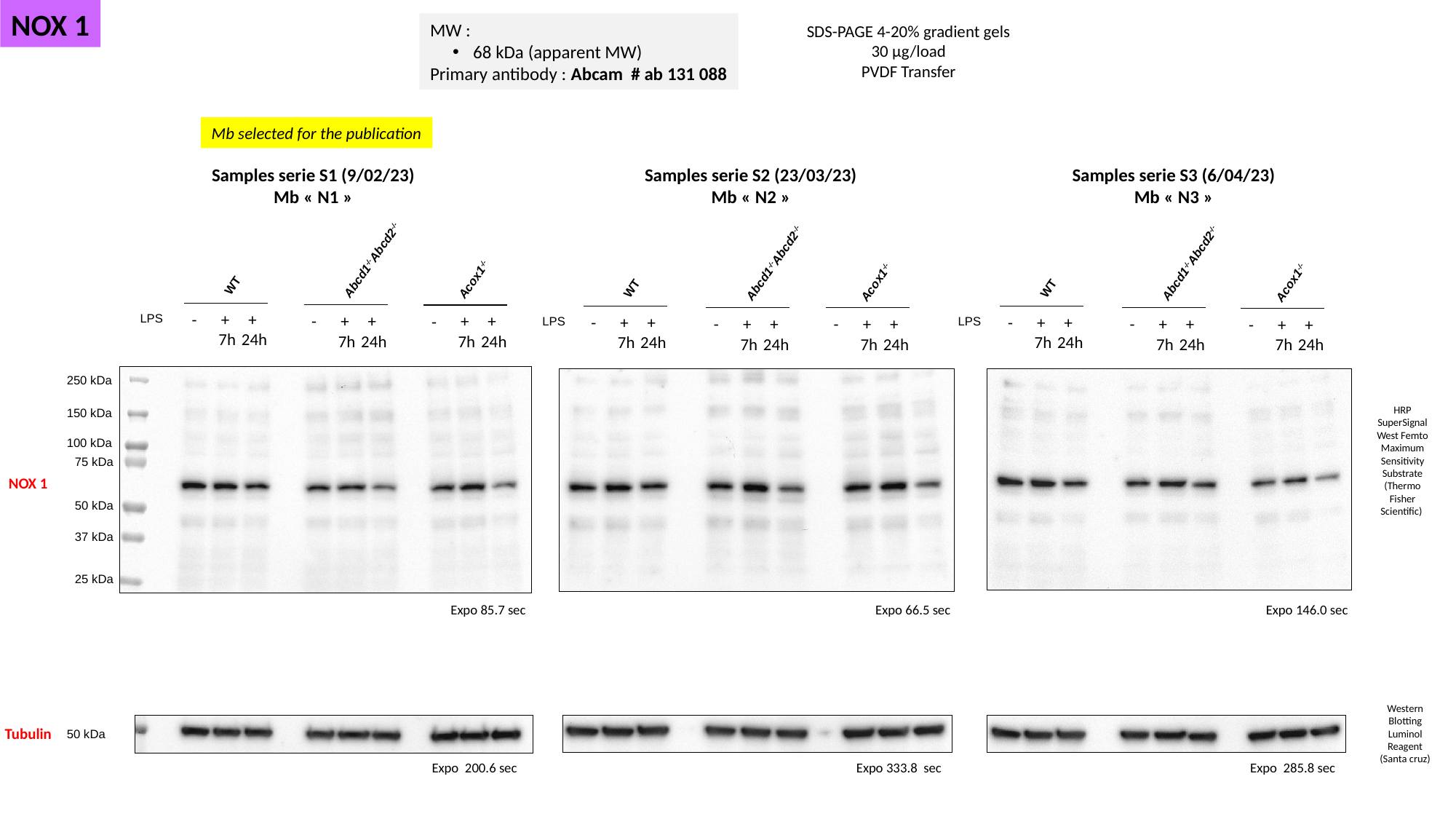

NOX 1
MW :
68 kDa (apparent MW)
Primary antibody : Abcam # ab 131 088
SDS-PAGE 4-20% gradient gels
30 µg/load
PVDF Transfer
Mb selected for the publication
Samples serie S1 (9/02/23)
Mb « N1 »
Samples serie S2 (23/03/23)
Mb « N2 »
Samples serie S3 (6/04/23)
Mb « N3 »
Abcd1-/-Abcd2-/-
-
+
7h
+
24h
Abcd1-/-Abcd2-/-
-
+
7h
+
24h
Abcd1-/-Abcd2-/-
-
+
7h
+
24h
Acox1-/-
Acox1-/-
Acox1-/-
WT
WT
WT
-
+
7h
+
24h
-
+
7h
+
24h
LPS
-
+
7h
+
24h
-
+
7h
+
24h
-
+
7h
+
24h
-
+
7h
+
24h
LPS
LPS
250 kDa
150 kDa
100 kDa
75 kDa
50 kDa
37 kDa
25 kDa
HRP SuperSignal West Femto Maximum Sensitivity Substrate (Thermo Fisher Scientific)
NOX 1
Expo 85.7 sec
Expo 66.5 sec
Expo 146.0 sec
Western Blotting Luminol Reagent (Santa cruz)
Tubulin
50 kDa
Expo  200.6 sec
Expo 333.8  sec
Expo  285.8 sec

## Slide 6
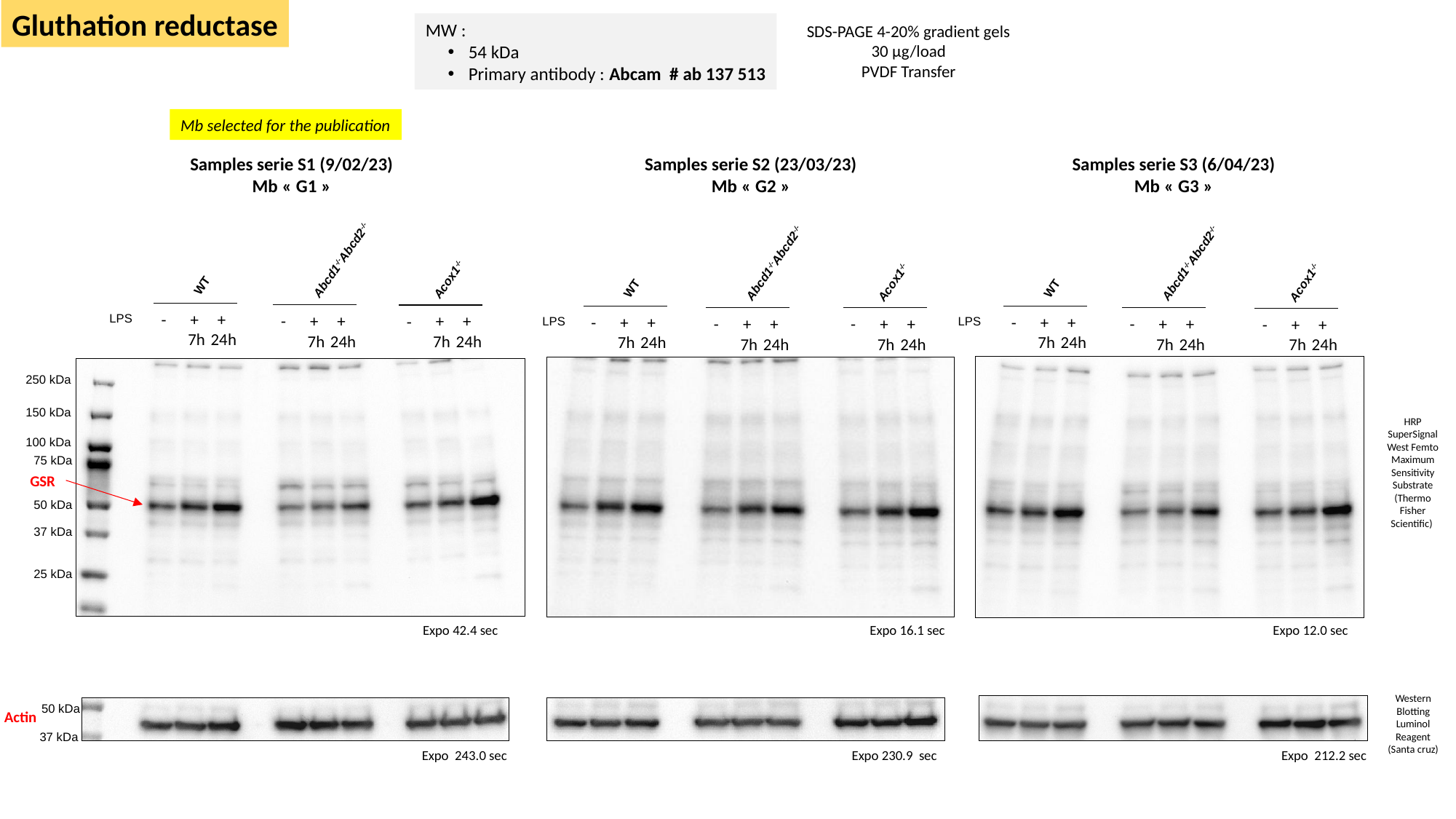

Gluthation reductase
MW :
54 kDa
Primary antibody : Abcam # ab 137 513
SDS-PAGE 4-20% gradient gels
30 µg/load
PVDF Transfer
Mb selected for the publication
Samples serie S1 (9/02/23)
Mb « G1 »
Samples serie S2 (23/03/23)
Mb « G2 »
Samples serie S3 (6/04/23)
Mb « G3 »
Abcd1-/-Abcd2-/-
-
+
7h
+
24h
Abcd1-/-Abcd2-/-
-
+
7h
+
24h
Abcd1-/-Abcd2-/-
-
+
7h
+
24h
Acox1-/-
Acox1-/-
Acox1-/-
WT
WT
WT
-
+
7h
+
24h
-
+
7h
+
24h
LPS
-
+
7h
+
24h
-
+
7h
+
24h
-
+
7h
+
24h
-
+
7h
+
24h
LPS
LPS
250 kDa
150 kDa
100 kDa
75 kDa
50 kDa
37 kDa
25 kDa
HRP SuperSignal West Femto Maximum Sensitivity Substrate (Thermo Fisher Scientific)
GSR
Expo 42.4 sec
Expo 16.1 sec
Expo 12.0 sec
Western Blotting Luminol Reagent (Santa cruz)
50 kDa
Actin
37 kDa
Expo  243.0 sec
Expo 230.9  sec
Expo  212.2 sec

## Slide 7
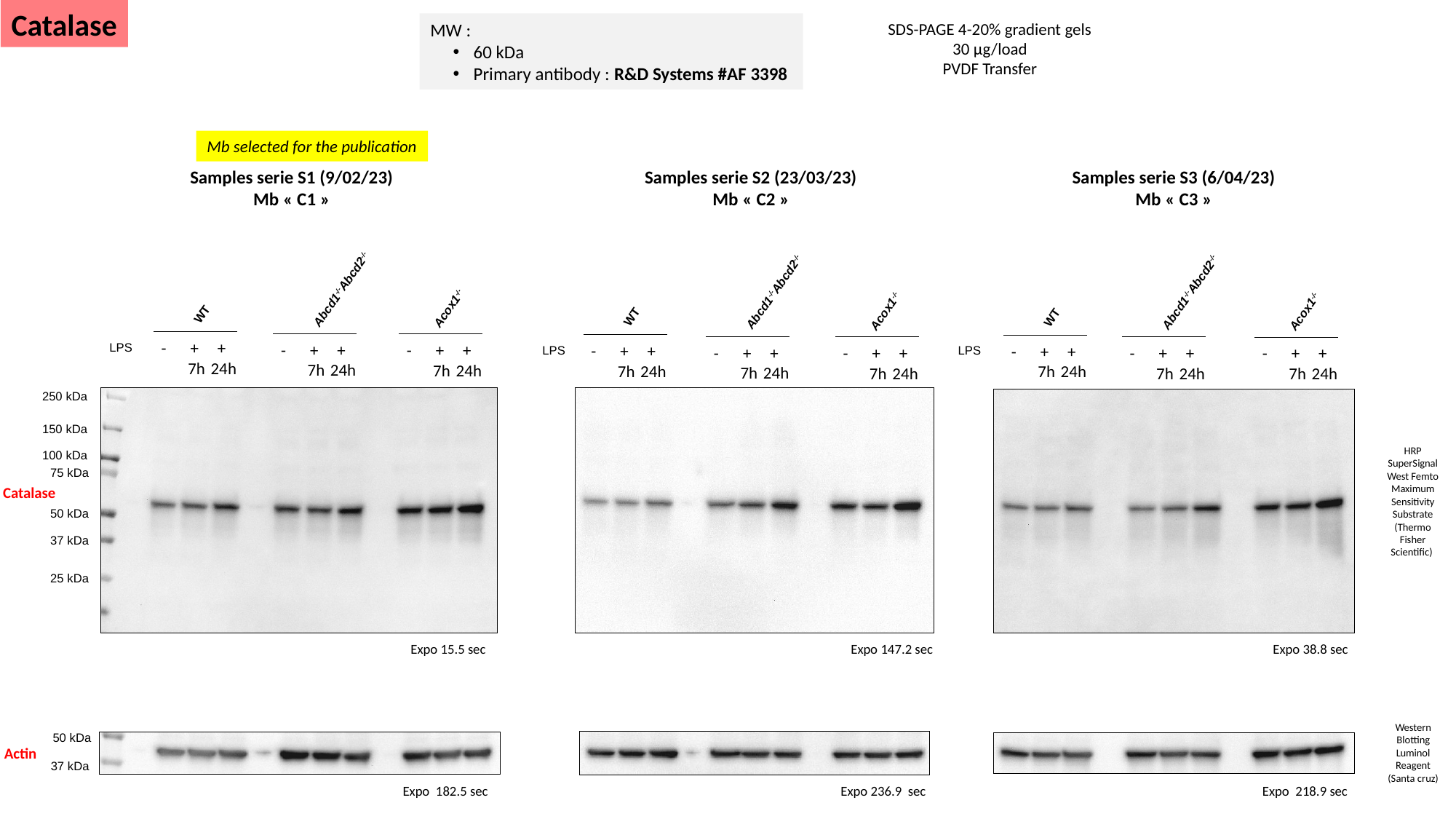

Catalase
SDS-PAGE 4-20% gradient gels
30 µg/load
PVDF Transfer
MW :
60 kDa
Primary antibody : R&D Systems #AF 3398
Mb selected for the publication
Samples serie S1 (9/02/23)
Mb « C1 »
Samples serie S2 (23/03/23)
Mb « C2 »
Samples serie S3 (6/04/23)
Mb « C3 »
Abcd1-/-Abcd2-/-
-
+
7h
+
24h
Abcd1-/-Abcd2-/-
-
+
7h
+
24h
Abcd1-/-Abcd2-/-
-
+
7h
+
24h
Acox1-/-
Acox1-/-
Acox1-/-
WT
WT
WT
-
+
7h
+
24h
-
+
7h
+
24h
LPS
-
+
7h
+
24h
-
+
7h
+
24h
-
+
7h
+
24h
-
+
7h
+
24h
LPS
LPS
250 kDa
150 kDa
100 kDa
75 kDa
50 kDa
37 kDa
25 kDa
HRP SuperSignal West Femto Maximum Sensitivity Substrate (Thermo Fisher Scientific)
Catalase
Expo 15.5 sec
Expo 147.2 sec
Expo 38.8 sec
Western Blotting Luminol Reagent (Santa cruz)
50 kDa
Actin
37 kDa
Expo  182.5 sec
Expo 236.9  sec
Expo  218.9 sec
